# Supplementary material for: NUDIX hydrolases target specific inositol pyrophosphates and regulate phosphate homeostasis and bacterial pathogen susceptibility in Arabidopsis
Source: J Integr Plant Biol. 2025 Oct 30;67(12):3123–51. doi: 10.1111/jipb.70060 (PMC12678693; doi:10.1111/jipb.70060)
Supplement: Supplementary file 1 — Figure S1. Structure of myo‐inositol and 5PCP‐InsP5 Figure S2. NUDT hydrolases from Subclades I and II exhibit differential expression in the ddp1Δ yeast strain Figure S3. NUDT17 does not hydrolyze InsP6 in vitro Figure S4. Arabidopsis NUDT hydrolases of Subclade I display 4‐InsP7 pyrophosphatase activity in vitro Figure S5. Subclade II NUDT hydrolases lose substrate specificity at higher concentrations in vitro Figure S6. Arabidopsis NUDT hydrolases of Subclade II display 3‐InsP7 pyrophosphatase activity in vitro Figure S7. Arabidopsis NUDT hydrolases show a weak hydrolysis activity toward 1,5‐InsP8 in vitro Figure S8. Arabidopsis NUDT hydrolases display differential hydrolysis activity toward 3,5‐InsP8 in vitro Figure S9. Subclade I NUDT hydrolases lose substrate specificity at higher concentrations in vitro Figure S10. Single and double knockout mutants do not show a significant (PP‐)InsP increase Figure S11. Schematic representation of the mutations in Subclade I nudt4/17/18/21 mutants Figure S12. Schematic representation of the mutations in Subclade II nudt12/13/16 mutants Figure S13. Transient expression of Nudix‐type (NUDT) hydrolases in Nicotiana benthamiana reveals 4/6‐InsP7 and 5‐InsP7 turnover in planta Figure S14. Transient co‐expression of the RUBY reporter with Subclade II Nudix‐type (NUDT) hydrolase genes under the transcriptional control of the viral CaMV 35S promoter Figure S15. Differential gene expression and Gene Ontology (GO) enrichment analyses of nudt12/13/16 triple mutant Figure S16. 3PP‐InsP species are preferred substrates for evolutionarily conserved kinase and ADP phosphotransfer reactions Figure S17. Fungal NUDT hydrolase effectors show similar substrate specificities as Subclade I NUDTs Figure S18. Expression analysis of NUDT hydrolase genes in nematode‐infected plants and disease susceptibility assays of nudt12/nudt13/nudt16 lines with Hyaloperonospora arabidopsidis Table S1. List of known and putative Arabidopsis PP‐InsP interactors a [file JIPB-67-3123-s001.docx]

# Supporting Information


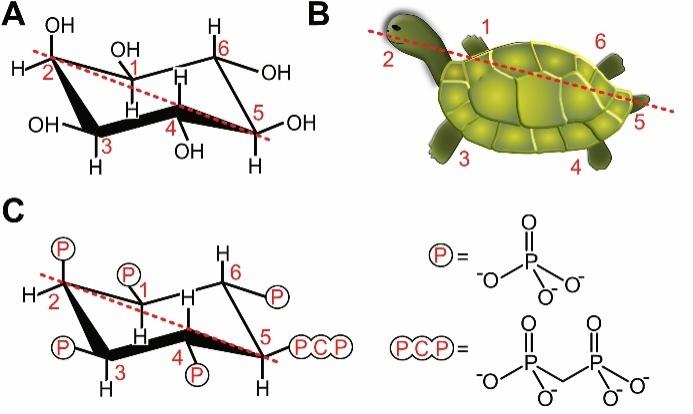


Figure S1: Structure of *myo*-inositol and 5PCP-InsP_5_. **(A)** Spatial representation of *myo*-inositol as chair conformation. **(B)** Schematic representation of *myo*-inositol based on Agranoff’s turtle. **(C)** Spatial representation of 5PCP-InsP_5_ as chair conformation.


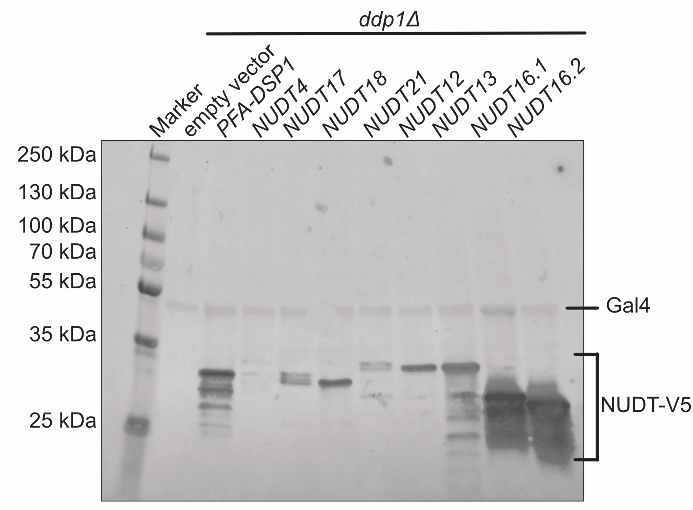


Figure S2: Nudix-type (NUDT) hydrolases from Subclades I and II exhibit differential expression in the *ddp1Δ* yeast strain. Immunoblot analysis was performed on protein extracts from ddp1Δ yeast transformed with either an empty pAG425-GPD-*ccdB* plasmid, a pAG425 carrying PFA-DSP1 (used as a positive control for the V5 tag), or pAG425 constructs encoding NUDTs fused to a C-terminal V5 tag. Detection of V5-tagged proteins was carried out using an anti-V5 primary antibody (Invitrogen; 1:2,000 dilution) and an Alexa Fluor Plus 800-conjugated anti-mouse secondary antibody (Invitrogen, goat; 1:20,000 dilution). Gal4 protein levels served as a loading control and were simultaneously detected using a polyclonal anti-Gal4 antibody (Santa Cruz; 1:1,000 dilution) along with a StarBright Blue 700-conjugated anti-rabbit secondary antibody (Bio-Rad, goat; 1:2,500 dilution). Signals were visualized using the multiplex mode of the ChemiDoc MP imaging system (Bio-Rad).


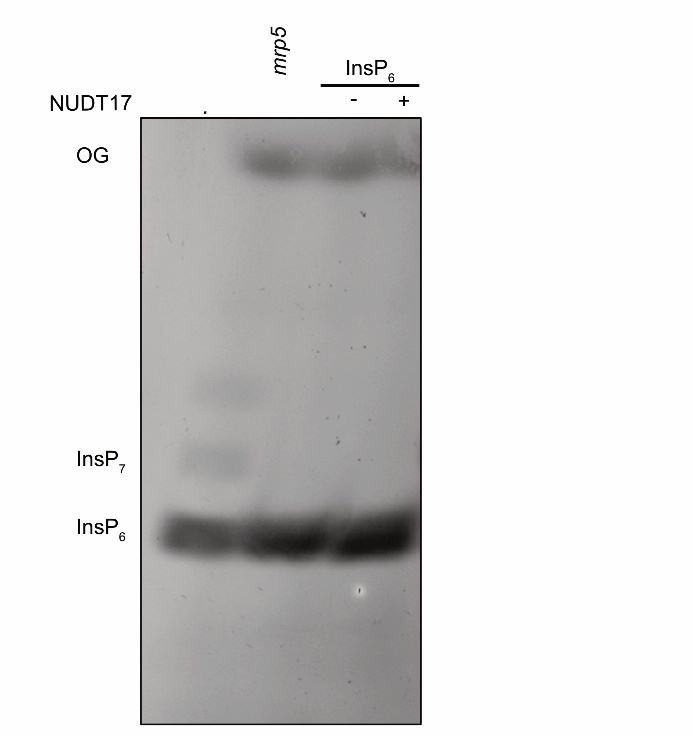


Figure S3: NUDT17 does not hydrolyze InsP_6_ *in vitro*. Recombinant His_6_-MBP-NUDT17 was incubated with 0.33 mM InsP_6_ and 1 mM MgCl_2_ at 28°C. Recombinant His_8_-MBP served as a negative control (indicated with the minus). After 1 h, the reactions were analyzed by 33% PAGE and visualized by toluidine blue staining. OG: orange G.


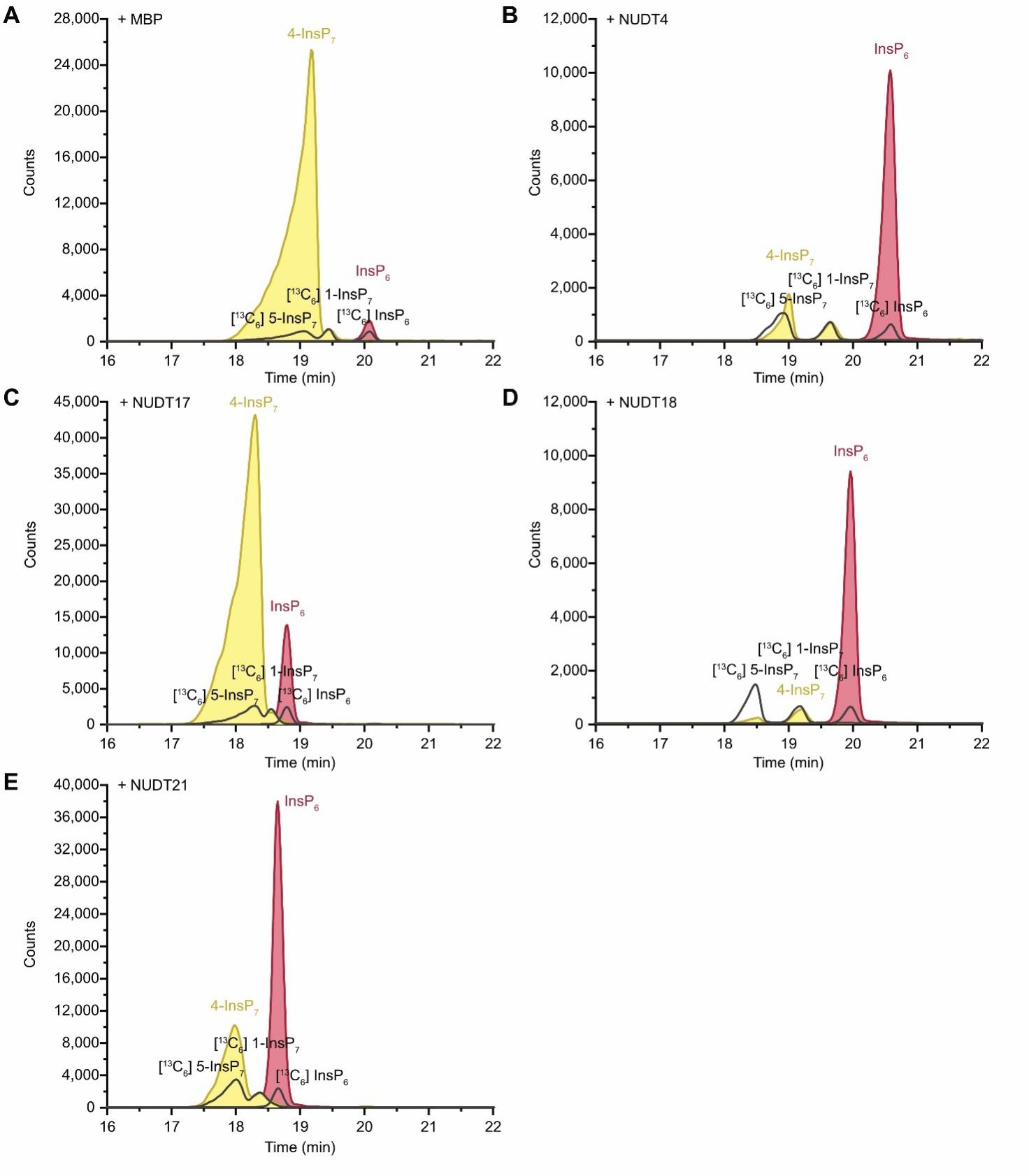


Figure S4: Arabidopsis Nudix-type (NUDT) hydrolases of Subclade I display 4-InsP_7_ pyrophosphatase activity *in vitro*. Recombinant His_8_-MBP (negative control, **A**), and His_6_-MBP-NUDT4 **(B)**, -17 **(C)**, -18 **(D)**, or -21 **(E)** were incubated with 0.33 mM 4‑InsP_7_ and 1 mM MgCl_2_ at 28°C for 1 h. Reactions were terminated by freezing at -80°C and stored until analysis. Prior to CE-ESI-MS analysis, samples were spiked with an isotopic standard mix ([^13^C_6_] 1,5-InsP_8_, [^13^C_6_] 5-InsP_7_, [^13^C_6_] 1-InsP_7_, [^13^C_6_] InsP_6_, [^13^C_6_] 2-OH InsP_5_).


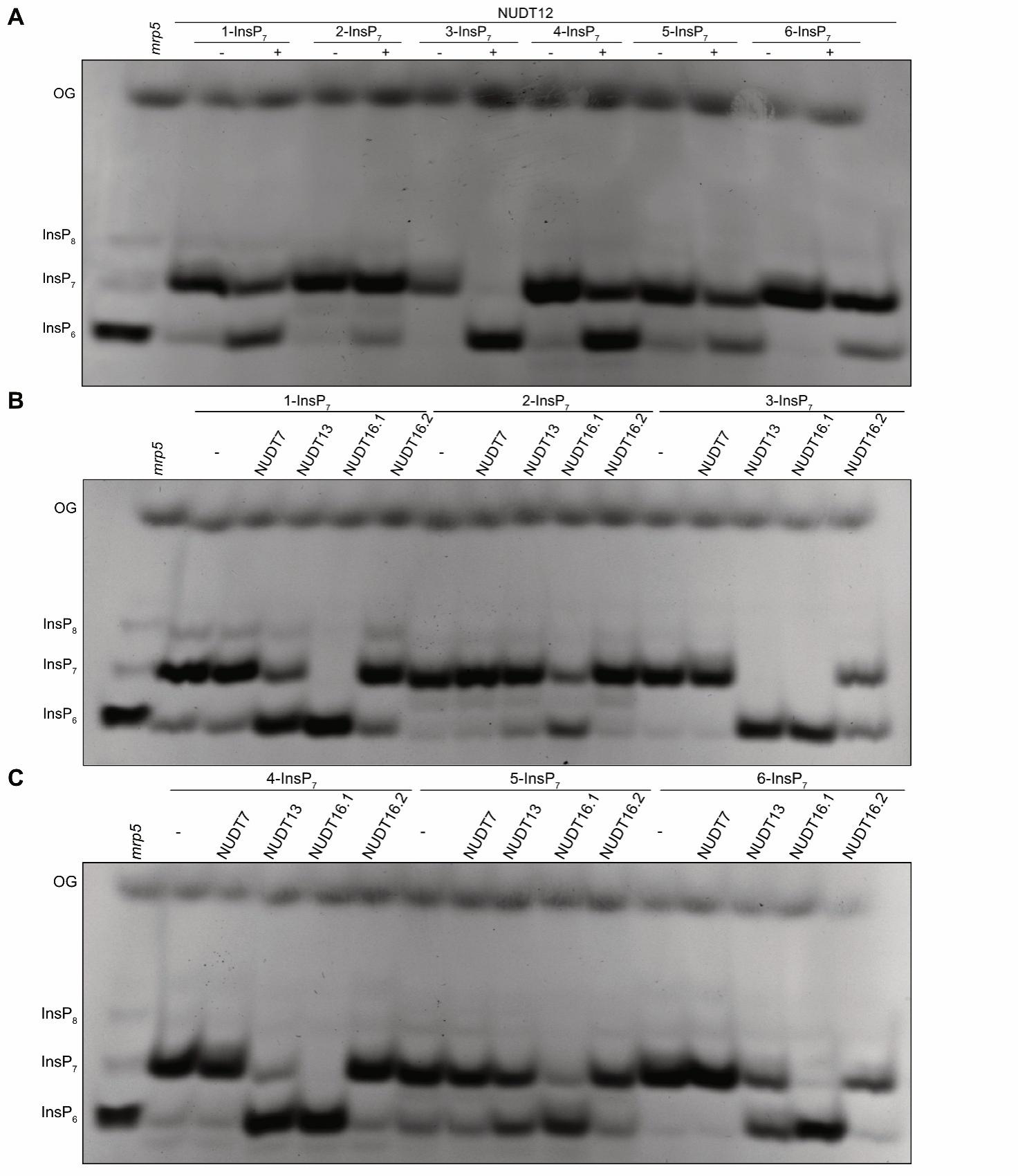


Figure S5: Subclade II Nudix-type (NUDT) hydrolases lose substrate specificity at higher concentrations *in vitro*. Recombinant His_6_-MBP-tagged NUDT12 (∼7.5 µM, **A**), NUDT7 (∼7 µM), NUDT13 (∼7.5 µM), NUDT16.1 (∼8 µM) or NUDT16.2 (∼8 µM) (**B** and **C**) were each incubated with 0.33 mM InsP_7_ and 1 mM MgCl_2_ at 28°C. Plus signal in **(A)** indicates presence of NUDT12 in the reaction. His_8_-MBP served as a negative control (indicated with the minus symbol in all panels). After 1 h, the reaction products were separated by 33% PAGE and visualized by toluidine blue. OG: orange G.


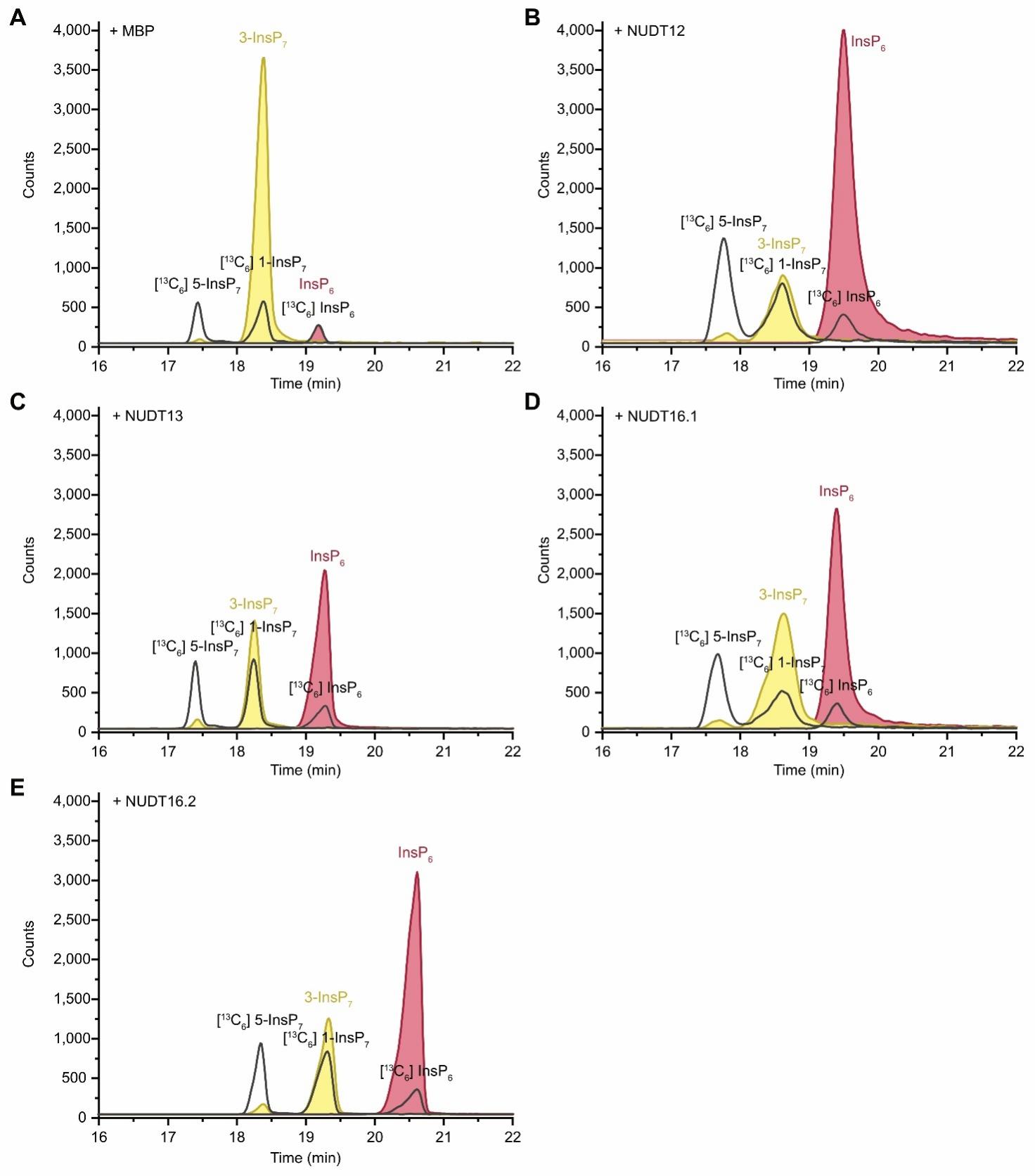


Figure S6: Arabidopsis Nudix-type (NUDT) hydrolases of Subclade II display 3-InsP_7_ pyrophosphatase activity *in vitro*. Recombinant His_8_-MBP (negative control, **A**) and His_6_-MBP-NUDT12 **(B)**, ‑13 **(C)**, ‑16.1 **(D)**, or -16.2 **(E)** were incubated with 0.33 mM 3-InsP_7_ and 1 mM MgCl_2_ at 28°C for 1 h. Reactions were terminated by freezing at -80°C and stored until analysis. Prior to CE-ESI-MS analysis, samples were spiked with an isotopic standard mix ([^13^C_6_] 1,5‑InsP_8_, [^13^C_6_] 5-InsP_7_, [^13^C_6_] 1-InsP_7_, [^13^C_6_] InsP_6_, [^13^C_6_] 2‑OH InsP_5_).


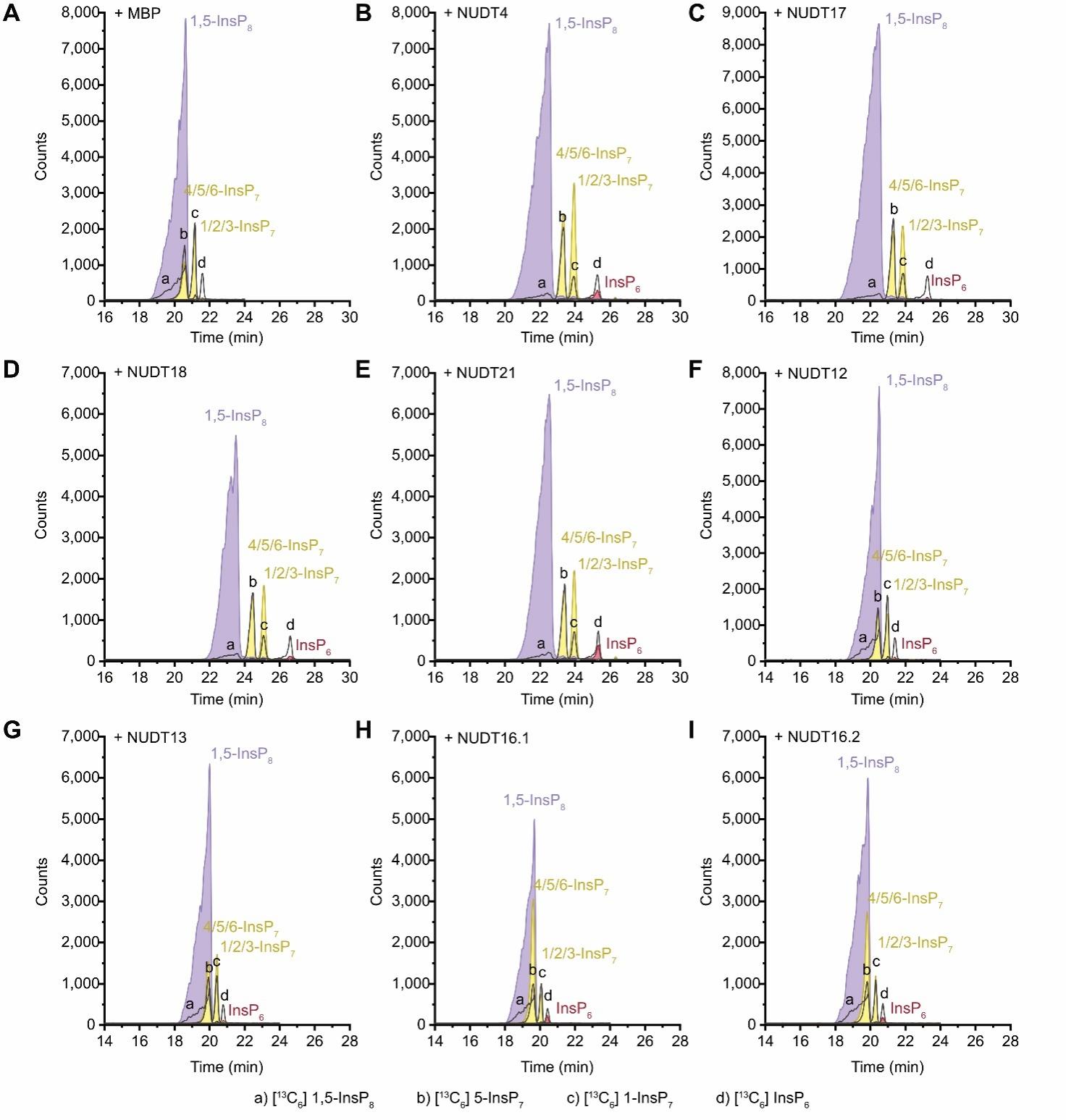


Figure S7: Arabidopsis Nudix-type (NUDT) hydrolases show a weak hydrolysis activity toward 1,5‑InsP_8_ *in vitro*. Recombinant His_8_-MBP (negative control, **A**) and His_6_-MBP-NUDT proteins **(B-I)** were incubated with 0.33 mM 1,5‑InsP_8_ and 1 mM MgCl_2_ at 28°C for 1 h. Reactions were terminated by freezing at -80°C and stored until analysis. Prior to CE-ESI-MS analysis, samples were spiked with an isotopic standard mix ([^13^C_6_] 1,5‑InsP_8_, [^13^C_6_] 5‑InsP_7_, [^13^C_6_] 1‑InsP_7_, [^13^C_6_] InsP_6_, [^13^C_6_] 2‑OH InsP_5_).


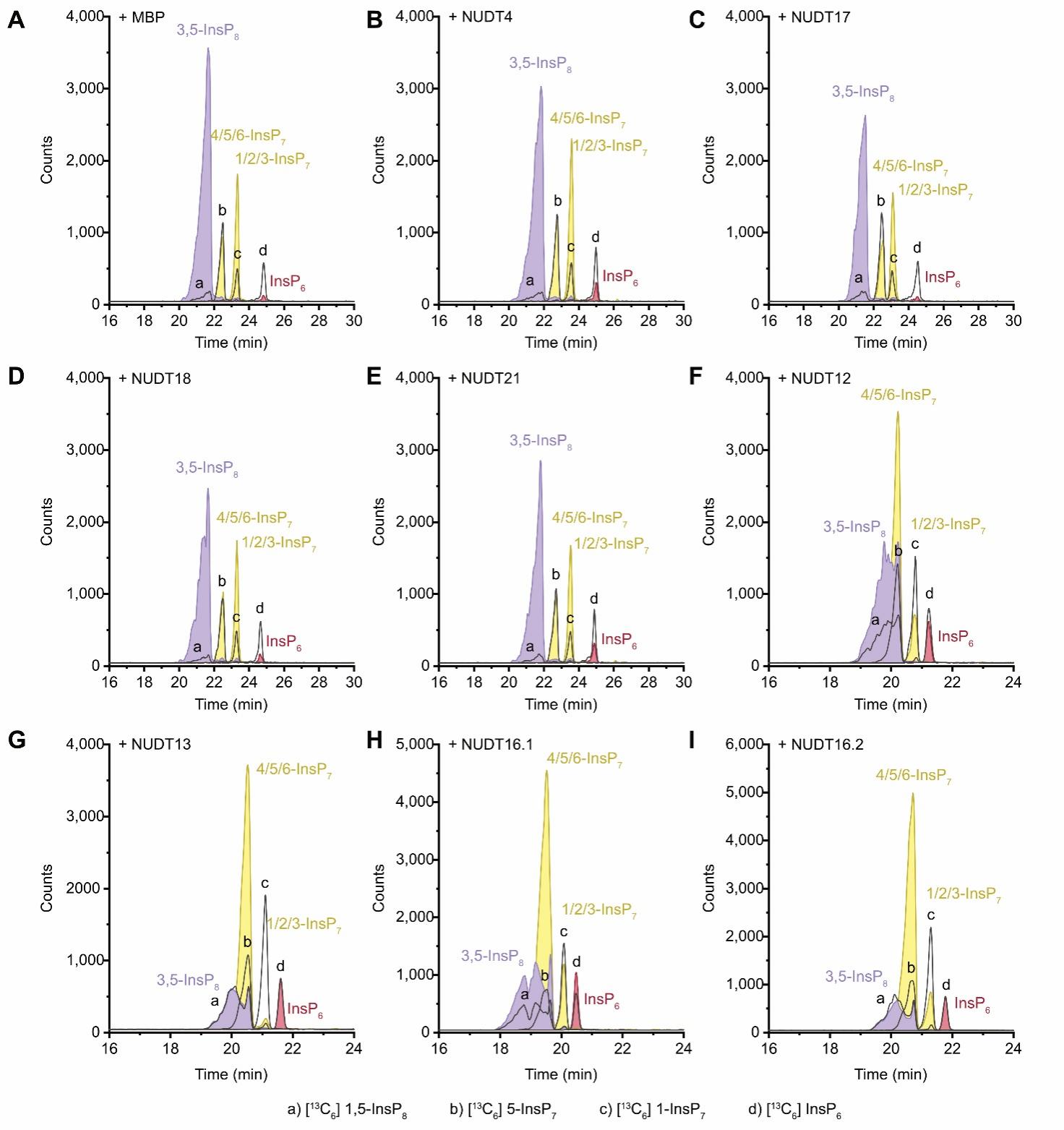


Figure S8: Arabidopsis Nudix-type (NUDT) hydrolases display differential hydrolysis activity toward 3,5‑InsP_8_ *in vitro*. Recombinant His_8_-MBP (negative control, **A**) and His_6_-MBP-NUDT proteins **(B-I)** were incubated with 0.33 mM 3,5‑InsP_8_ and 1 mM MgCl_2_ at 28°C for 1 h. Reactions were terminated by freezing at -80°C and stored until analysis. Prior to CE-ESI-MS analysis, samples were spiked with an isotopic standard mix ([^13^C_6_] 1,5‑InsP_8_, [^13^C_6_] 5‑InsP_7_, [^13^C_6_] 1-InsP_7_, [^13^C_6_] InsP_6_, [^13^C_6_] 2-OH InsP_5_).

*
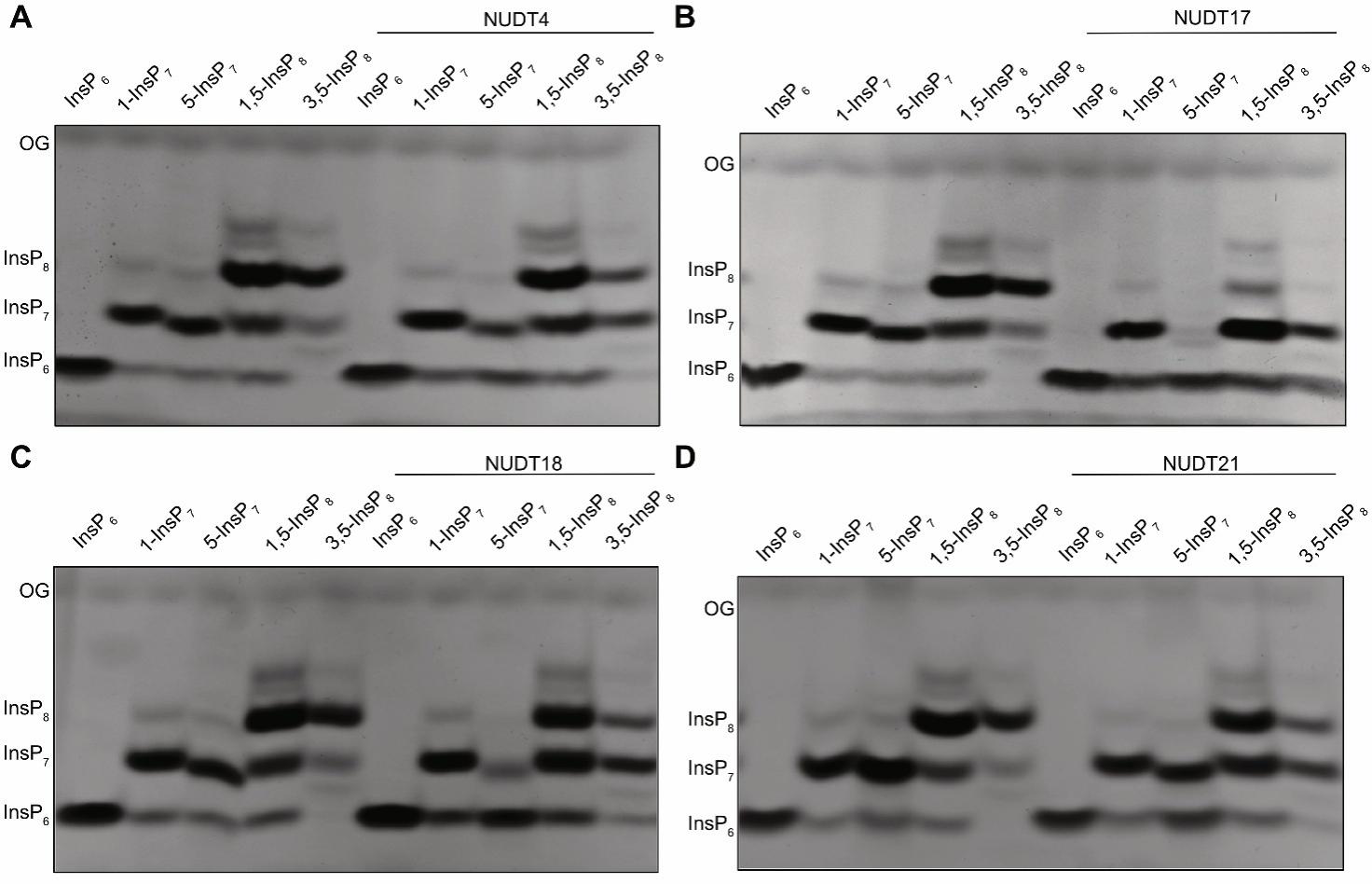
*

Figure S9: Subclade I Nudix-type (NUDT) hydrolases lose substrate specificity at higher concentrations *in vitro*. Recombinant His_6_-MBP-tagged NUDT4 (∼9 µM, **A**), NUDT17 (∼6 µM, **B**), NUDT18 (∼6 µM, **C**) or NUDT21 (∼8 µM, **D**) were each incubated with 0.33 mM InsP_6_, InsP_7_ or InsP_8_, and 1 mM MgCl_2_ at 22°C. His_8_-MBP served as a negative control (placed at the left half of each gel). After 2 h, the reaction products were separated by 33% PAGE and visualized by toluidine blue. OG: orange G.


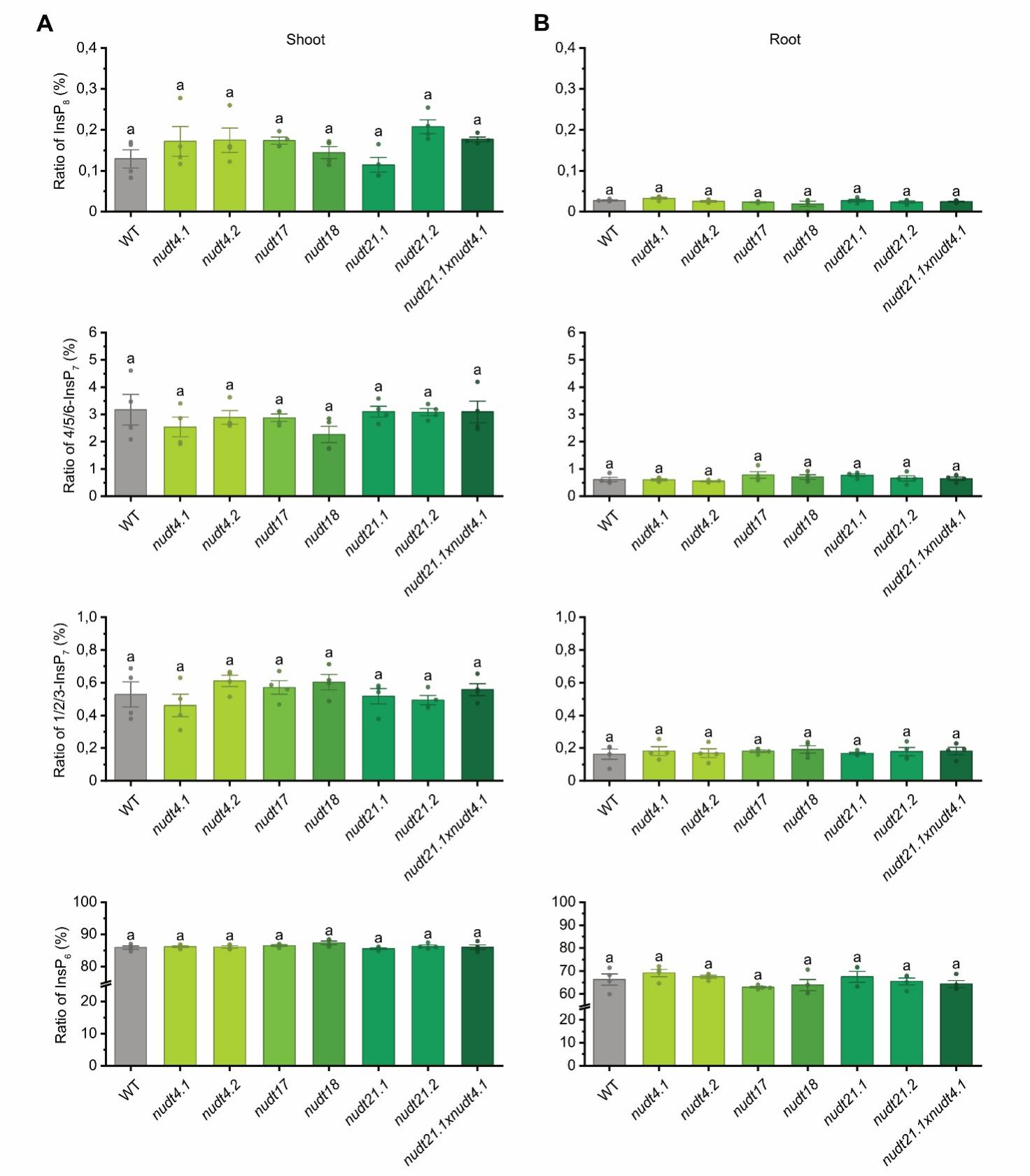


Figure S10: Single and double knockout mutants do not show a significant (PP-)InsP increase. *Arabidopsis thaliana* T-DNA insertion lines were grown hydroponically for 5 weeks. (PP-)InsPs of shoots **(A)** and roots **(B)** were purified with TiO_2_ and stored at -80°C until analysis. Prior to CE-ESI-MS analysis, samples were spiked with an isotopic standard mix ([^13^C_6_] 1,5‑InsP_8_, [^13^C_6_] 5‑InsP_7_, [^13^C_6_] 1‑InsP_7_, [^13^C_6_] InsP_6_, [^13^C_6_] 2‑OH InsP_5_). Different letters indicate values that are significantly different determined with one-way ANOVA followed by Dunn-Šidák test with a significance level of 0.05.


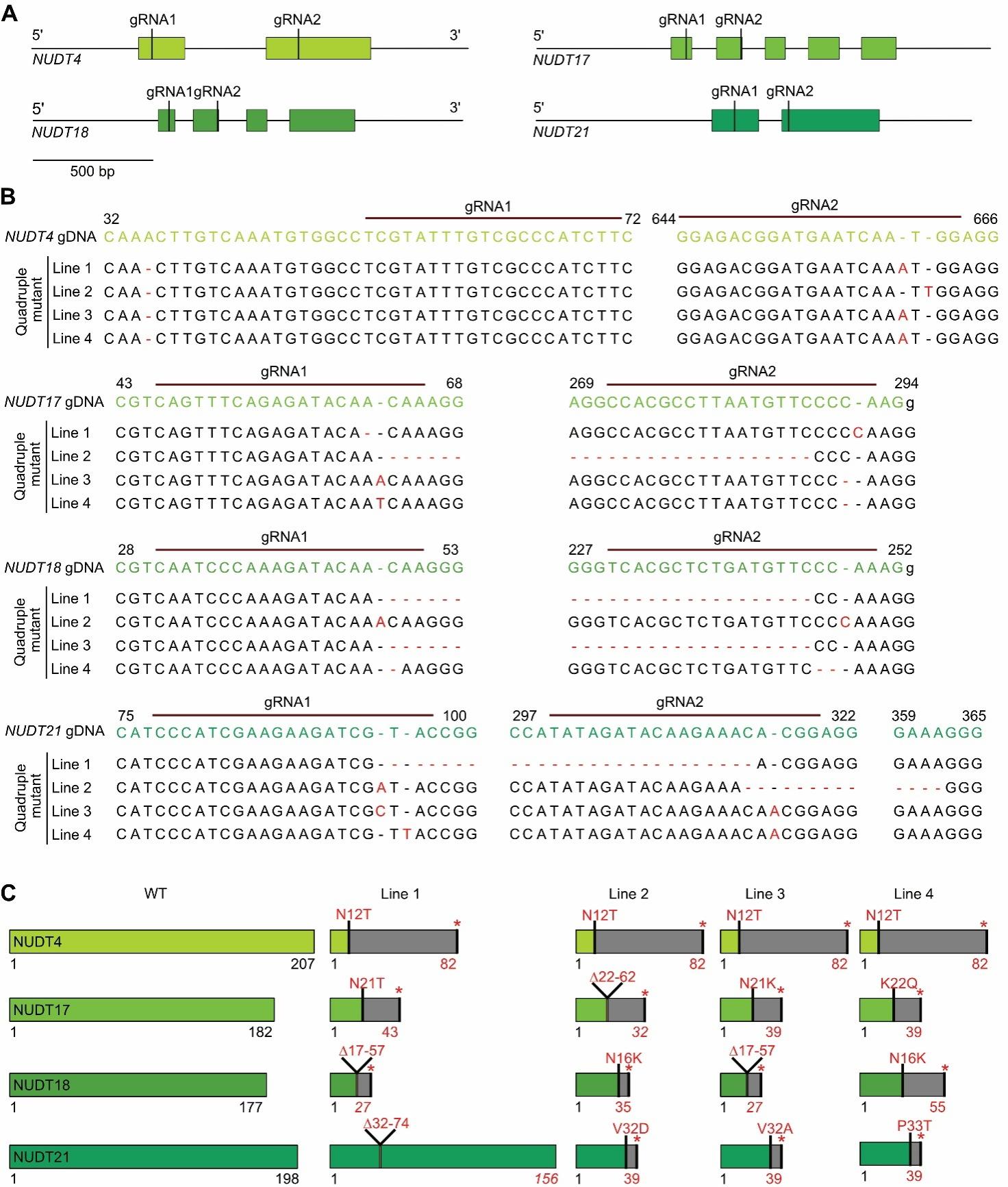


Figure S11: Schematic representation of the mutations in Subclade I *nudt4/17/18/21* mutants. **(A)** gRNA1 was designed to target the beginning of the open reading frame (ORF), while gRNA2 was designed to target the sequence encoding the Nudix-type (NUDT) motif (GX_5_EX_7_REUXEEXGU, where U represents a hydrophobic amino acid such as leucine, isoleucine or valine and X represents any amino acid), or its flanking region. **(B)** Observed deletions, insertions and point mutations in four *nudt4/17/18/21* independent mutant lines. **(C)** Predicted amino acids changes caused by respective mutations.


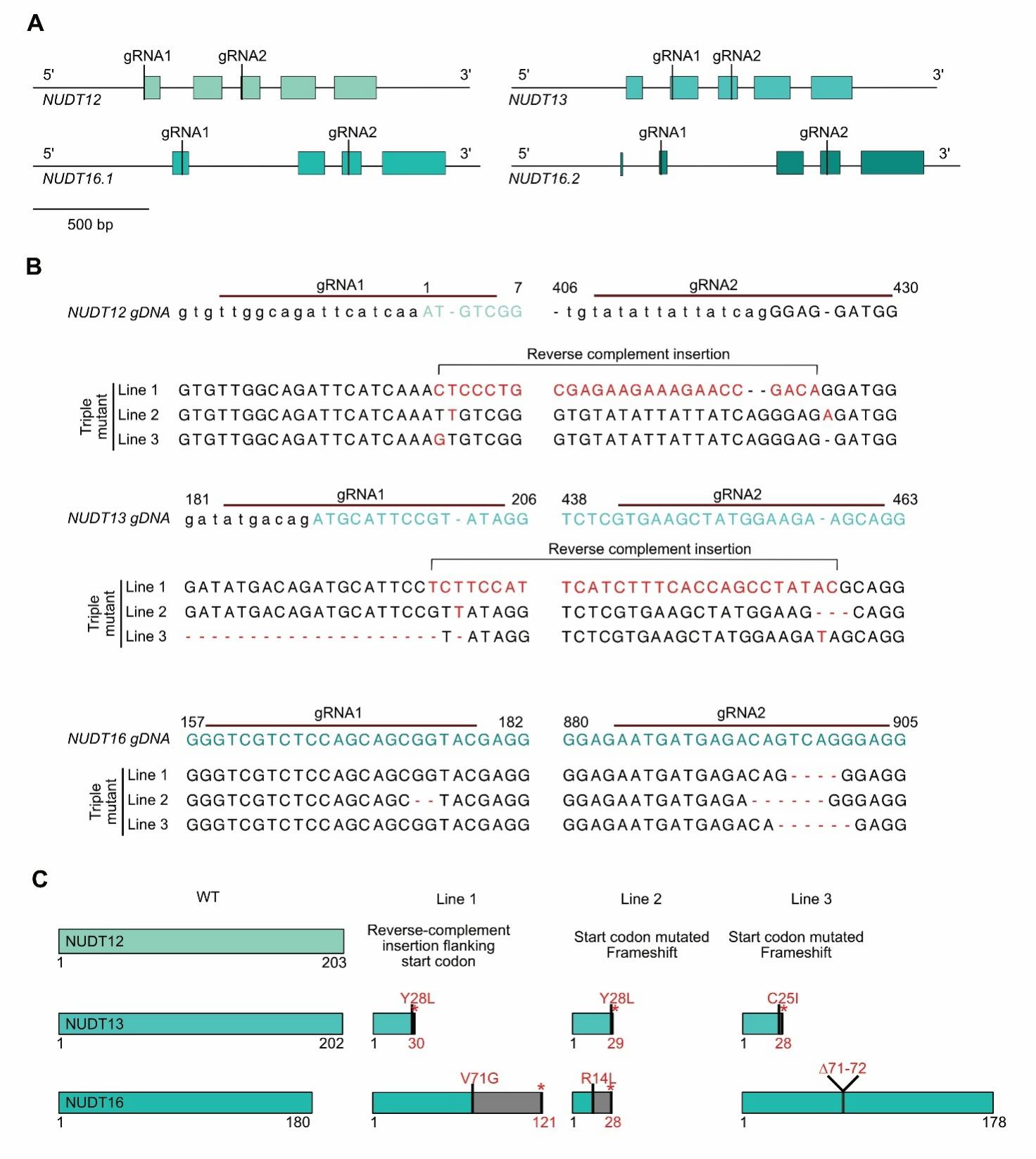


Figure S12: Schematic representation of the mutations in Subclade II *nudt12/13/16* mutants. **(A)** gRNA1 was designed to target the beginning of the open reading frame (ORF), while gRNA2 was designed to target the sequence encoding the Nudix-type (NUDT) motif (GX_5_EX_7_REUXEEXGU, where U represents a hydrophobic amino acid such as leucine, isoleucine or valine and X represents any amino acid), or its flanking region. **(B)** Observed deletions, insertions and point mutations in three *nudt12/13/16* mutants. **(C)** Predicted amino acids changes caused by respective mutations.


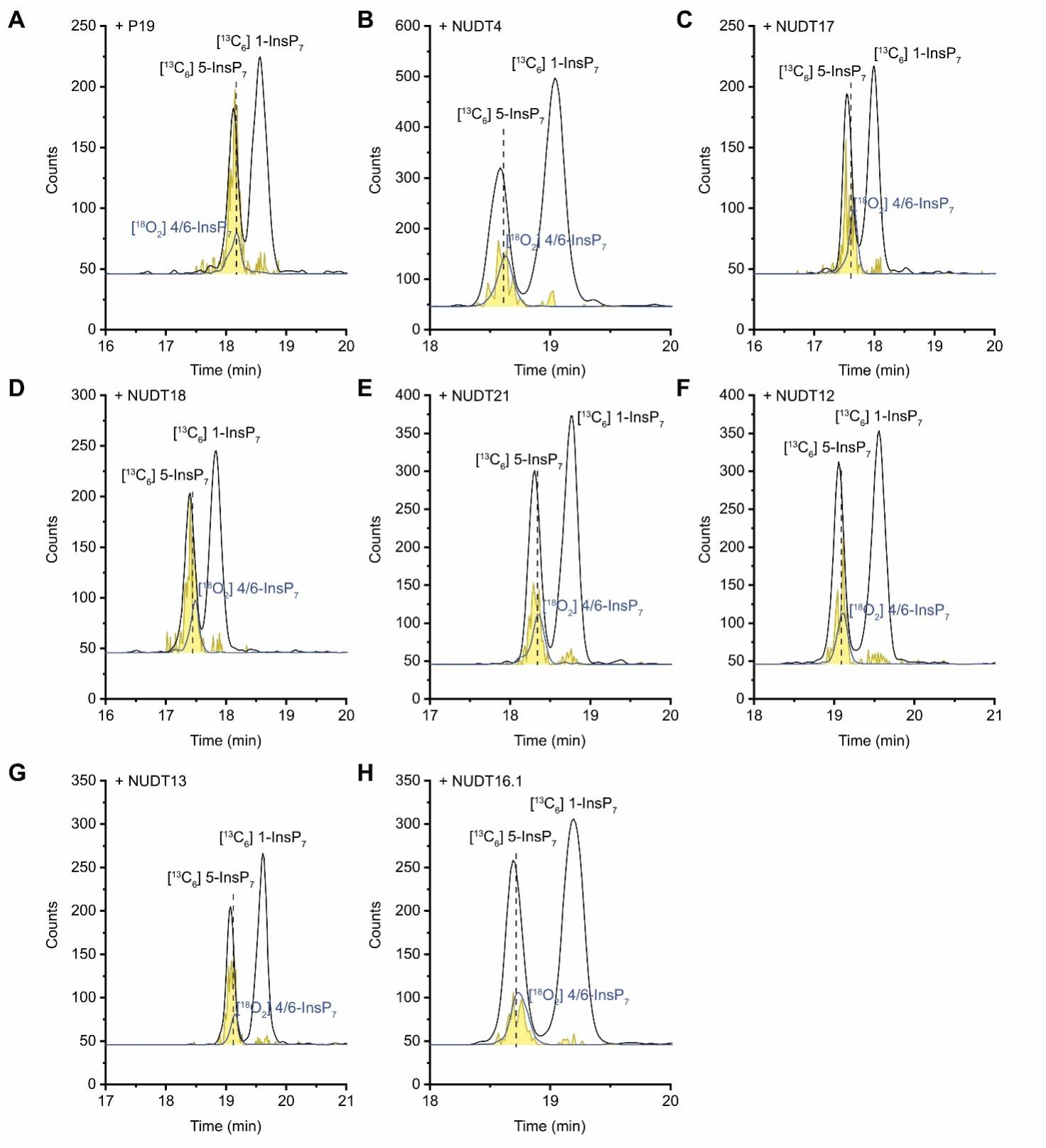


Figure S13: Transient expression of *Nudix-type* (*NUDT)* hydrolases in *Nicotiana benthamiana* reveals 4/6‑InsP_7_ and 5‑InsP_7_ turnover *in planta*. The silencing inhibitor P19 alone **(A)** or together with *NUDTs* **(B-H)** was transiently expressed in *N. benthamiana* leaves. 2-3 days post infiltration (dpi) (PP-)InsPs were purified with Nb_2_O_5_ beads, spiked with isotopic standard mix ([^13^C_6_] 1,5-InsP_8_, [^13^C_6_] 5‑InsP_7_, [^18^O_2_] 4‑InsP_7_, [^13^C_6_] 1‑InsP_7_, [^13^C_6_] InsP_6_, [^13^C_6_] 2‑OH InsP_5_) and subjected to CE-ESI-MS analyses.


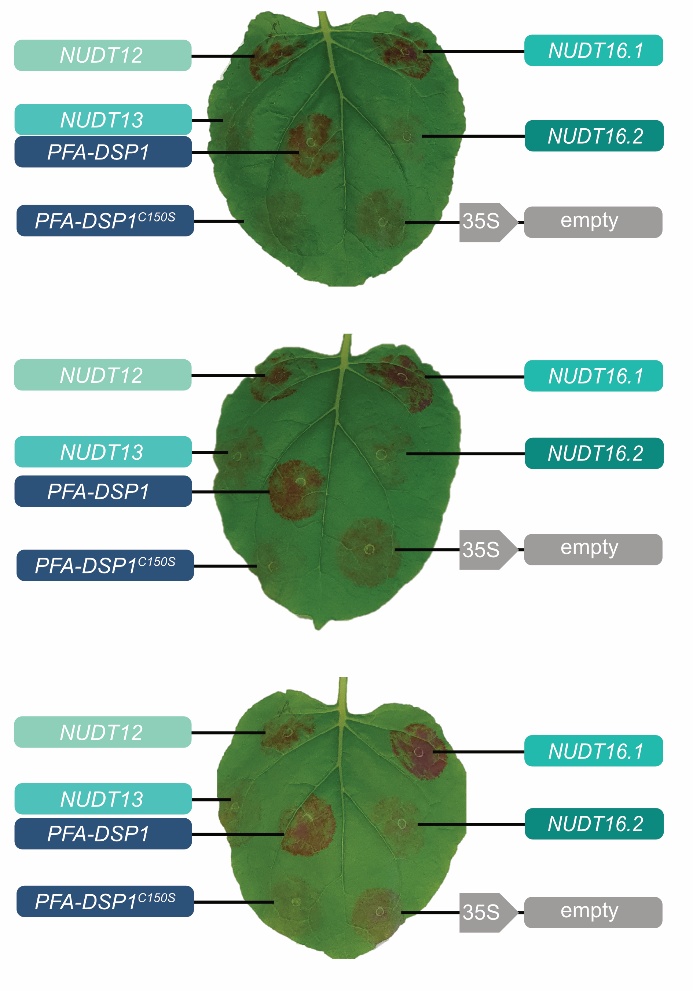


Figure S14: Transient co-expression of the *RUBY* reporter with subclade II *Nudix-type* (*NUDT)* hydrolase genes under the transcriptional control of the viral CaMV 35S promoter. Co-expression with *PFA-DSP1* served as a positive control, while co-expression with the catalytic inactive *PFA‑DSP1^C150S^* encoding the catalytic inactive protein or an empty vector served as negative controls. The picture was taken 3 days post infiltration.


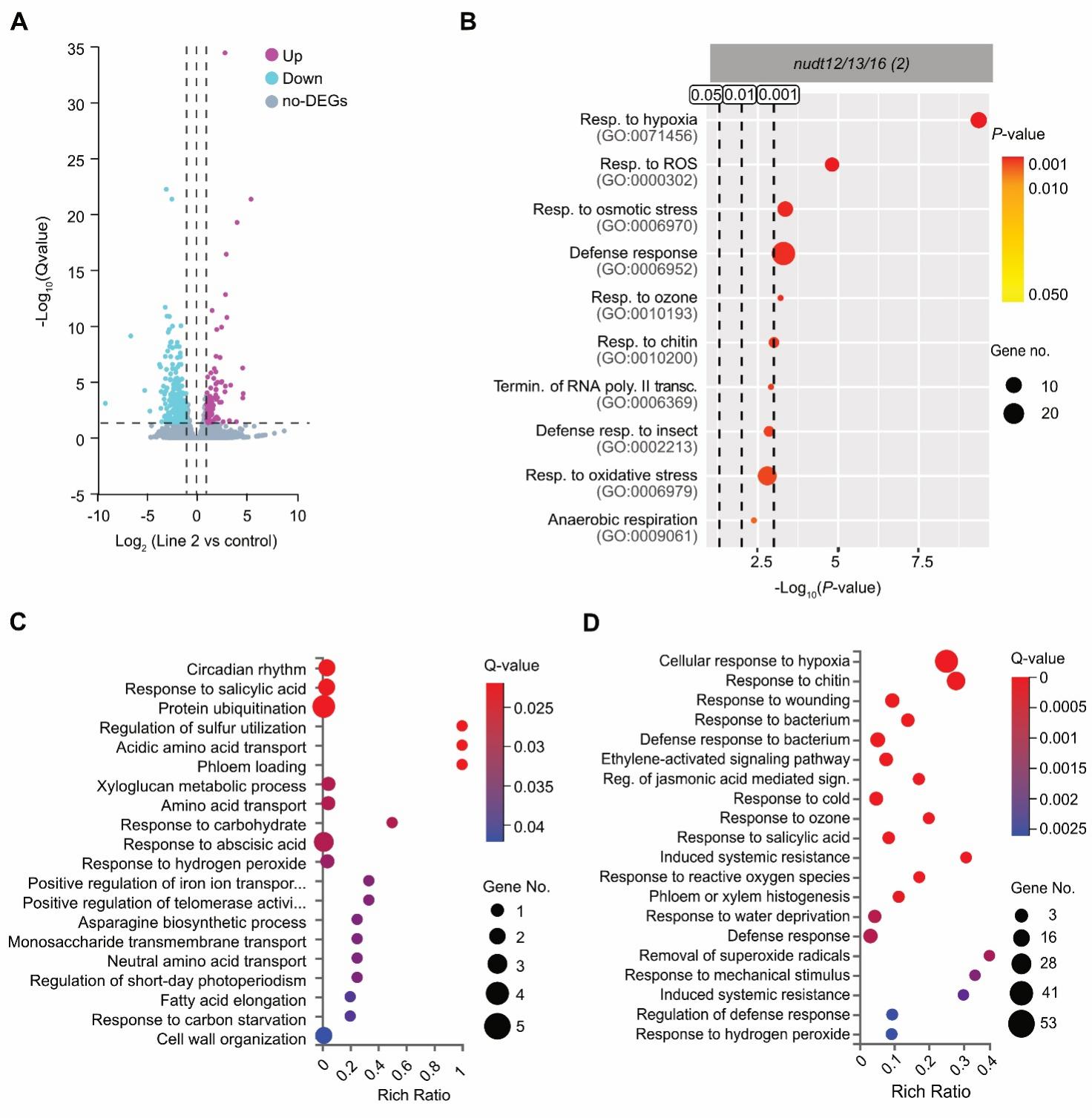


Figure S15: Differential gene expression and Gene Ontology (GO) enrichment analyses of *nudt12/13/16* triple mutant. **(A)** Volcano plot comparing *nudt12/13/16* mutant line 2 to wild type (WT). The x‑axis represents the log_2_FC values, and the y-axis shows -log_10_‑transformed significance values. Magenta dots indicate upregulated differentially expressed genes (DEGs), cyan dots indicate downregulated DEGs, and gray dots indicate non-DEGs. Gene Ontology enrichment analysis **(B)** suggests that Subclade II Nudix-type (NUDT)-dependent PP‑InsPs regulate PSR-unrelated processes with numerous genes involved in plant defense and related GO terms. Shown is a GO enrichment analysis of DEGs with Q < 0.05 and (|log_2_FC| > 1), based on Biological Processes for *nudt12/13/16* triple mutant line 2. The x-axis represents the statistical significance of these GO terms in ‑log_10_ (*P*-value), while the y-axis lists the GO terms with their respective ID. Bubble size indicates the number of DEGs annotated to each GO term, and the graph highlights three significance thresholds: 0.05, 0.01, and 0.001. The color of the bubbles corresponds to the *P*‑value, with red indicating more significant enrichment. GO enrichment bubble chart of DEGs for **(C)** upregulated and **(D)** downregulated genes with Q < 0.05 and (|log_2_FC| > 1), based on Biological Processes for triple mutant lines. The x-axis represents the enrichment ratio of genes, and the y-axis represents the GO term. The size of the bubble represents the number of differential genes annotated to a certain GO term. The color represents the significance value of enrichment (Q-value), where red indicates smaller significance values. ROS: reactive oxygen species, poly.: polymerase, transc.: transcription, reg.: regulation, sign.: signaling.


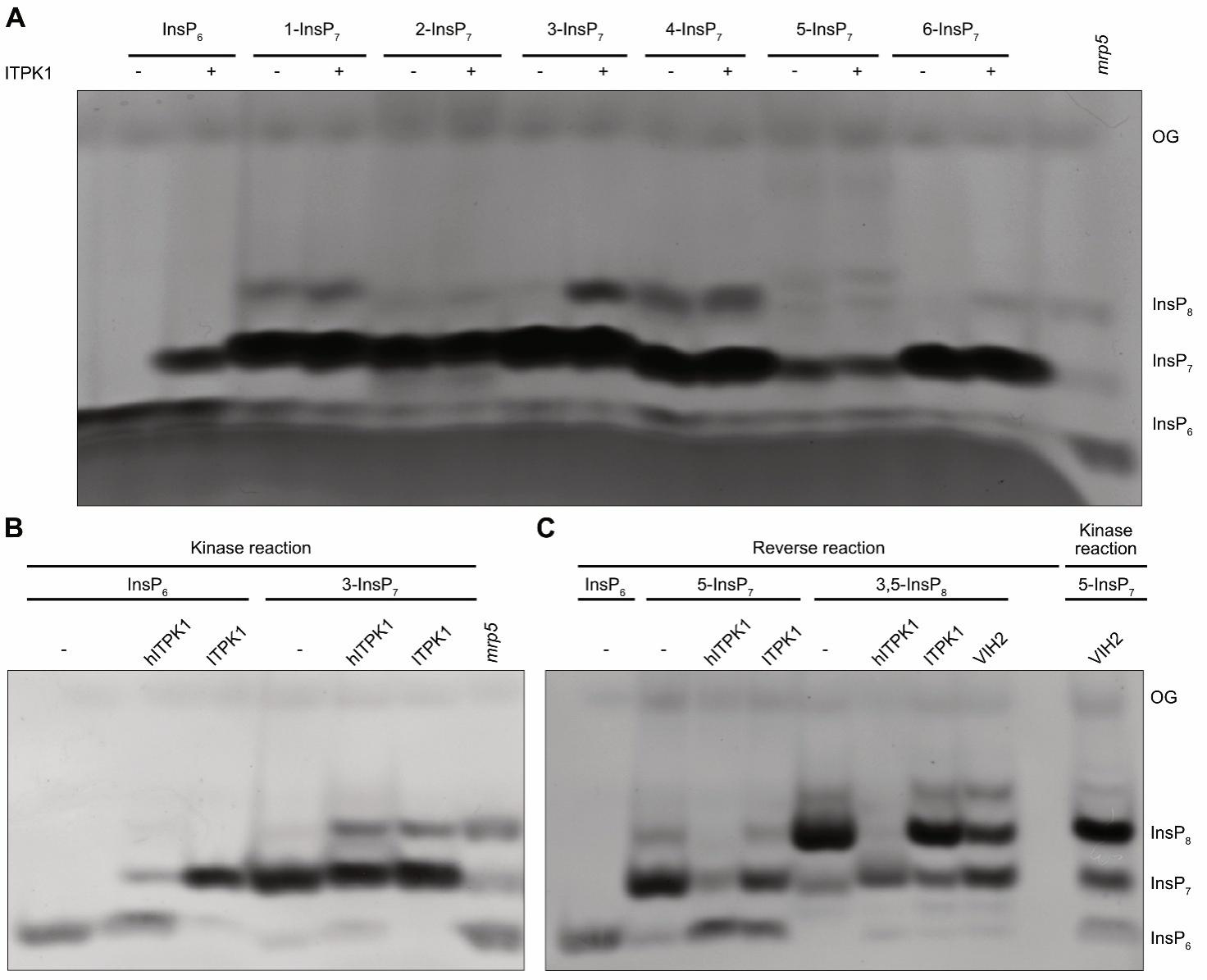


Figure S16: 3PP-InsP species are preferred substrates for evolutionarily conserved kinase and ADP phosphotransfer reactions. **(A)** Kinase assay with recombinant His_8_-MBP-ITPK1 and InsP_6_ or InsP_7_ species as indicated. **(B)** Kinase assay (kinase reaction) and PP-InsP/ADP phosphotransfer assay (reverse reaction, **C**) with recombinant His_8_-MBP-ITPK1, His_8_-MBP-VIH2 and His_8_-MBP-hsITPK1 and 1 mM of the indicated (PP-)InsP species. **(A, B)** After 6 h at 25°C the reactions were terminated by freezing at -80°C and stored until the reaction products were separated by 33% PAGE. (PP-)InsPs were visualized by toluidine blue staining. His_8_-MBP served as a negative control (indicated with the minus symbol). KD: kinase domain, OG: Orange G.


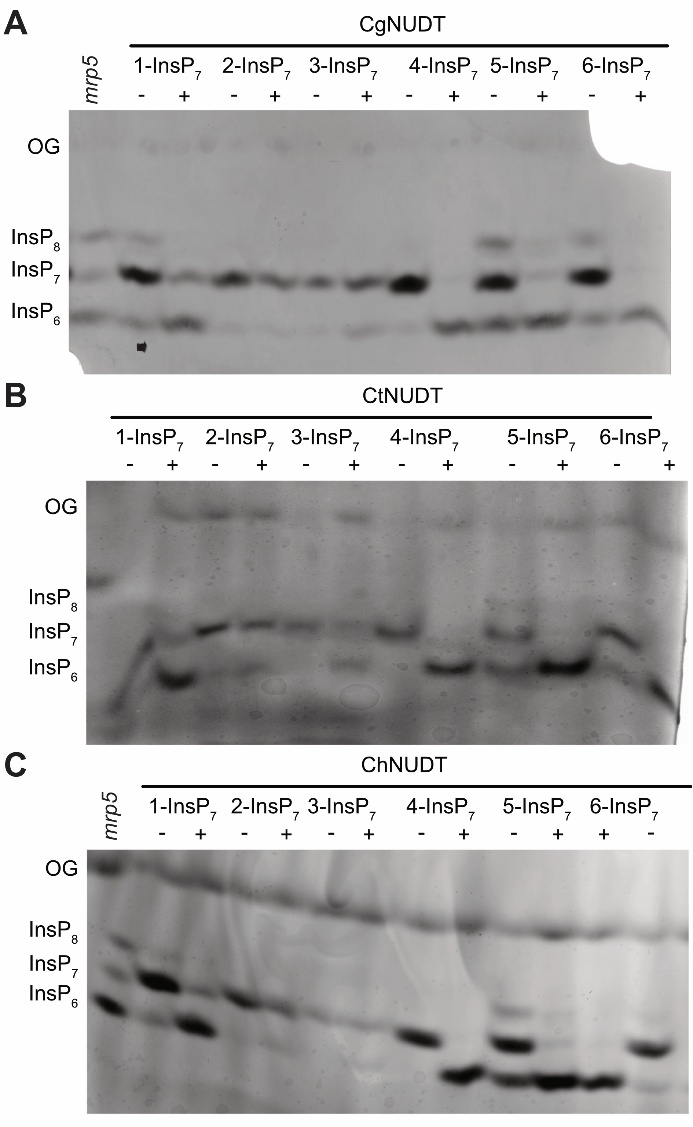


Figure S17: Fungal Nudix-type (NUDT) effectors show similar substrate specificities as Subclade I NUDTs. Recombinant His_6_-tagged CgNUDT **(A)**, CtNUDT **(B)**, or ChNUDT **(C)** were incubated with 0.25 mM InsP_7_. Lanes with a minus symbol show control reactions where no protein was added. After 45 min, reactions were terminated by freezing at -80°C and stored until the reaction products were separated by 33% PAGE. (PP-)InsPs were visualized by toluidine blue staining. A TiO_2_-purified Arabidopsis *mrp5* seed extract was used as a marker for InsP_6_, InsP_7_ and InsP_8_. OG: Orange G.


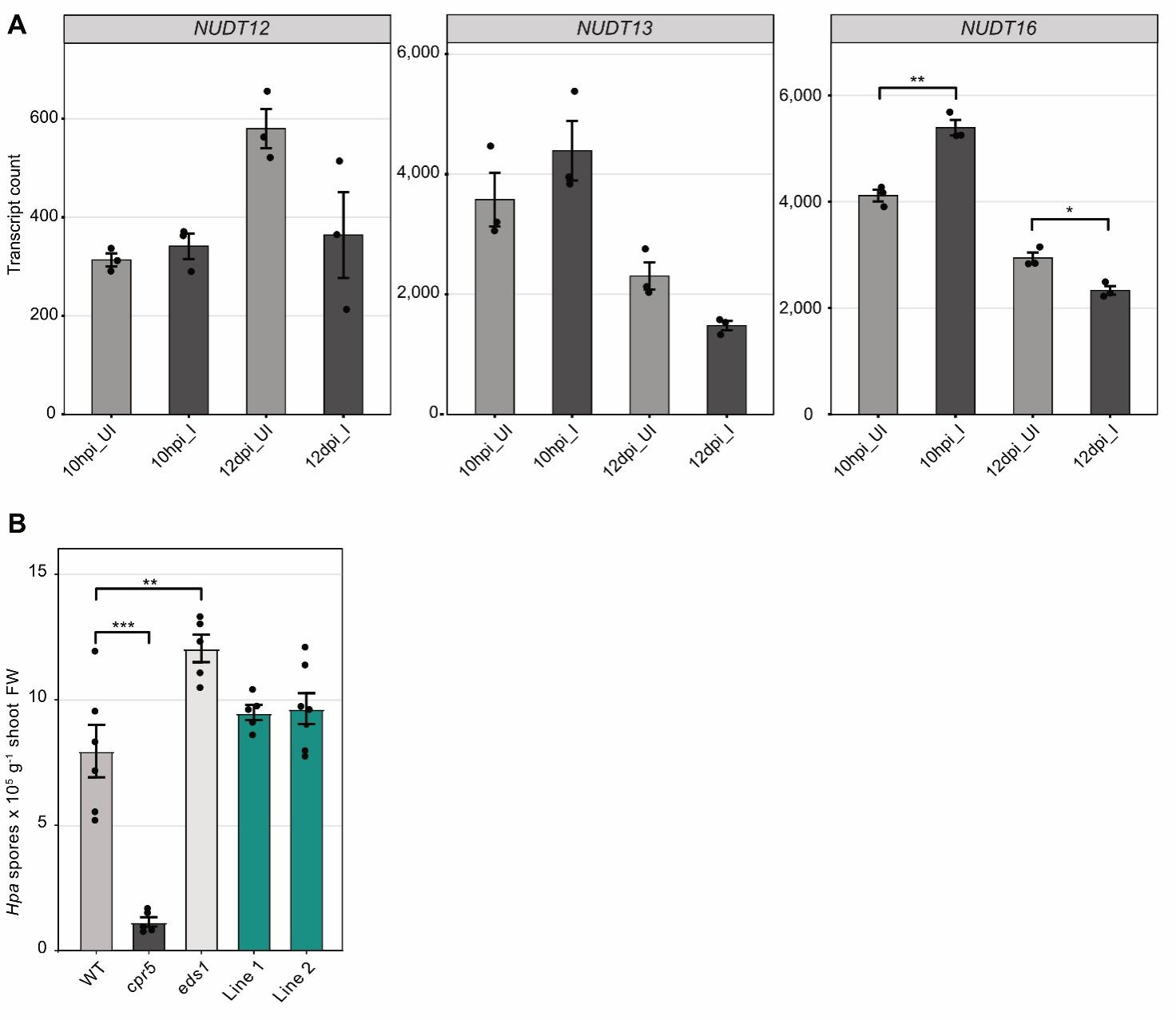


Figure S18: Expression analysis of Nudix-type (NUDT) hydrolase genes in nematode-infected plants and disease susceptibility assays of *nudt12/nudt13/nudt16* lines with *Hyaloperonospora arabidopsidis*. **(A)** Expression patterns of NUDT12, NUDT13, and NUDT16 based on RNA-Seq data from Siddique et al. (2022) hpi: hours post-inoculation; dpi: days post-inoculation; UI: uninfected root tissue; I: infected root tissue. Statistical analysis was performed using the Wilcoxon test for unpaired samples (*P* > 0.05; *P* ≤ 0.05 (*); *P* ≤ 0.01 (**); P ≤ 0.001 (***)). **(B)** Bar graphs represent the mean number of spores ± SEM per gram shoot fresh weight (FW) isolated from Arabidopsis wild-type (WT) plants or the indicated mutants 7 dpi with *Hyaloperonospora arabidopsidis* isolate Noco2. *n* = 5. Asterisks indicate significant differences to WT determined by an ordinary one-way ANOVA with Dunnett's multiple comparisons test (*P* < 0.01 (**); *P* < 0.001 (***)).

Table S1:

List of known and putative Arabidopsis PP-InsP interactors and Nudix-type (NUDT) hydrolases identified via 5PCP-InsP_5_ affinity pull-down. Numbers indicate identified peptides. Grouping of genes indicates that identified peptides derived from either of those two proteins.

|  |  | **Shoot** | | **Root** | |
| --- | --- | --- | --- | --- | --- |
| **Gene Name** | **Accession Number** | **Beads** | **Eluate** | **Beads** | **Eluate** |
| AFB1 | AT4G03190.1 | 8 | 20 | 9 | 20 |
| AFB2 | AT3G26810.1 | 5 | 3 | 1 | 2 |
| AFB3 | AT1G12820.1 | 6 | 3 | 1 | 1 |
| ASK1 | AT1G75950.1 | 5 | 9 | 4 | 10 |
| ASK2 | AT5G42190.1 | 0 | 7 | 0 | 7 |
| COI1 | AT2G39940.1 | 6 | 10 | 1 | 3 |
| CSN1 | AT3G61140.1 | 1 | 2 | 2 | 1 |
| CSN2 | AT2G26990.1 | 0 | 0 | 2 | 0 |
| CSN4 | AT5G42970.1 | 6 | 7 | 5 | 4 |
| CSN5A | AT1G22920.1  AT1G22920.2 | 0 | 2 | 1 | 2 |
| CUL1 | AT4G02570.1  AT4G02570.2  AT4G02570.3  AT4G02570.4 | 4 | 1 | 1 | 0 |
| CUL2 | AT1G43140.1 | 2 | 0 | 1 | 0 |
| IPK2ɑ | AT5G07370.1  AT5G07370.2  AT5G07370.3  AT5G07370.4 | 0 | 0 | 3 | 5 |
| IPK2β | AT5G61760.1 | 0 | 2 | 4 | 3 |
| ITPK3 | AT4G08170.3 | 0 | 0 | 1 | 0 |
| MIPS3/MIPS1 | AT5G10170.1  AT4G39800.1 | 1 | 0 | 0 | 0 |
| NUDT16 | AT3G12600.1  AT3G12600.2 | 0 | 0 | 1 | 0 |
| NUDT17/NUDT18 | AT2G01670.1  AT1G14860.1 | 2 | 4 | 0 | 0 |
| NUDT18 | AT1G14860.1 | 0 | 0 | 0 | 1 |
| NUDT21/NUDT4 | AT1G73540.1  AT1G18300.1 | 5 | 7 | 0 | 0 |
| SPX1 | AT5G20150.1 | 1 | 0 | 0 | 0 |
| SPX2 | AT2G26660.1 | 0 | 0 | 2 | 0 |
| VIH1/VIH2 | AT5G15070.1  AT5G15070.2  AT3G01310.1  AT3G01310.2 | 4 | 4 | 13 | 7 |

Table S2: (PP-)InsP values in pmol g^-1^ FW for Arabidopsis grown on ½ Murashige–Skoog (½MS) plates.

|  | **InsP_8_** | **4/6-InsP_7_** | **5-InsP_7_** | **1/3-InsP_7_** | **InsP_6_** | **1/3OH-InsP_5_** | **InsP_5_ others** | **InsP_4-1_** | **InsP_4-2_** |
| --- | --- | --- | --- | --- | --- | --- | --- | --- | --- |
| **Line 1 *nudt12/13/16*** | 108 | 123 | 164 | 109 | 32987 | 1563 | 424 | 520 | 180 |
| **Line 1 *nudt12/13/16*** | 54 | 46 | 122 | 65 | 23653 | 1325 | nd | 286 | 105 |
| **Line 1 *nudt12/13/16*** | 62 | 48 | 89 | 63 | 21875 | 1362 | nd | 337 | 101 |
| **Line 1 *nudt12/13/16*** | 70 | 70 | 118 | 107 | 24237 | 1359 | 296 | 436 | 169 |
| **Line 1 *nudt12/13/16*** | 84 | 95 | 117 | 108 | 24482 | 1195 | 316 | 416 | 139 |
| **Line 2 *nudt12/13/16*** | 44 | 48 | 85 | 60 | 21334 | 1279 | nd | 282 | 107 |
| **Line 2 *nudt12/13/16*** | 19 | 34 | 81 | 39 | 15918 | 1034 | nd | 233 | 85 |
| **Line 2 *nudt12/13/16*** | 17 | 56 | 49 | 42 | 13666 | 554 | 152 | 176 | 70 |
| **Line 2 *nudt12/13/16*** | 40 | 61 | 90 | 53 | 22740 | 1339 | nd | 355 | 130 |
| **Line 2 *nudt12/13/16*** | 17 | 82 | 82 | 49 | 17076 | 825 | 158 | 207 | 51 |
| **Line 3 *nudt12/13/16*** | 116 | 106 | 121 | 161 | 35168 | 1585 | 517 | 678 | 217 |
| **Line 3 *nudt12/13/16*** | 84 | 68 | 111 | 102 | 26963 | 1398 | 512 | 500 | 156 |
| **Line 3 *nudt12/13/16*** | 32 | 89 | 141 | 99 | 27537 | 1769 | nd | 459 | 135 |
| **Line 3 *nudt12/13/16*** | 57 | 122 | 133 | 106 | 31685 | 1521 | 470 | 495 | 174 |
| **WT** | 44 | 86 | 75 | 19 | 26635 | 1088 | 307 | 398 | 110 |
| **WT** | 10 | 80 | 71 | 36 | 24818 | 1013 | 260 | 301 | 92 |
| **WT** | 15 | 122 | 75 | 50 | 25791 | 1072 | 286 | 408 | nd |
| **WT** | 36 | 73 | 65 | 15 | 29288 | 1203 | 384 | 587 | nd |
| **WT** | 23 | 67 | 95 | 18 | 30921 | 1380 | 416 | 550 | nd |
| **WT** | 9 | 88 | 76 | 15 | 28327 | 1093 | 308 | 428 | nd |
| **Line 2 *nudt4/17/18/21*** | 17 | 84 | 77 | 14 | 33146 | 1371 | 420 | 590 | nd |
| **Line 2 *nudt4/17/18/21*** | 26 | 82 | 81 | 41 | 26739 | 1199 | 333 | 490 | nd |
| **Line 2 *nudt4/17/18/21*** | 18 | 89 | 70 | 14 | 29768 | 1180 | 375 | 552 | nd |
| **Line 2 *nudt4/17/18/21*** | 57 | 109 | 103 | 74 | 37888 | 1620 | 483 | 710 | nd |
| **Line 2 *nudt4/17/18/21*** | 21 | 113 | 82 | 39 | 30777 | 1347 | 438 | 542 | nd |
| **Line 2 *nudt4/17/18/21*** | 13 | 77 | 64 | 13 | 29425 | 1193 | 345 | 539 | nd |
| **Line 3 *nudt4/17/18/21*** | 42 | 110 | 91 | 20 | 34357 | 1364 | 410 | 549 | nd |
| **Line 3 *nudt4/17/18/21*** | 21 | 54 | 57 | 15 | 27907 | 1129 | 289 | 377 | nd |
| **Line 3 *nudt4/17/18/21*** | 16 | 57 | 52 | 9 | 22202 | 949 | 209 | 333 | nd |
| **Line 3 *nudt4/17/18/21*** | 25 | 56 | 56 | 10 | 22985 | 1062 | 258 | 393 | nd |
| **Line 3 *nudt4/17/18/21*** | 62 | 120 | 110 | 49 | 35868 | 1445 | 407 | 540 | nd |
| **Line 3 *nudt4/17/18/21*** | 33 | 54 | 63 | 20 | 28096 | 1233 | 290 | 430 | nd |
| **Line4 *nudt4/17/18/21*** | 19 | 70 | 65 | 30 | 24263 | 1033 | 282 | 334 | nd |
| **Line4 *nudt4/17/18/21*** | 60 | 122 | 110 | 70 | 33129 | 1310 | 414 | 501 | nd |
| **Line4 *nudt4/17/18/21*** | 61 | 95 | 73 | 25 | 28582 | 434 | 363 | 493 | nd |
| **Line4 *nudt4/17/18/21*** | 193 | 133 | 135 | 101 | 37275 | 1704 | 546 | 785 | nd |
| **Line4 *nudt4/17/18/21*** | 29 | 82 | 80 | 52 | 28524 | 1076 | 276 | 415 | nd |
| **Line4 *nudt4/17/18/21*** | 46 | 54 | 68 | 11 | 29162 | 1193 | 338 | 454 | nd |

Table S3: (PP-)InsP values in pmol g^-1^ FW for transformed *Nicotiana benthamiana* plants.

|  | **InsP_8_** | **4/6-InsP_7_** | **5-InsP_7_** | **1/3-InsP_7_** | **InsP_6_** | **1/3OH-InsP_5_** | **InsP_5_ others** | **InsP_4-1_** | **InsP_4-2_** |
| --- | --- | --- | --- | --- | --- | --- | --- | --- | --- |
| **WT** | nd | 95 | 186 | 19 | 2381 | 88 | nd | 30 | 9 |
| **WT** | nd | 122 | 248 | 14 | 2851 | 200 | nd | 54 | 84 |
| **WT** | 31 | 48 | 115 | 15 | 1498 | 61 | 43 | 53 | 81 |
| **WT** | 40 | 117 | 137 | 15 | 2961 | 73 | 39 | 42 | 108 |
| **WT** | 45 | 130 | 175 | 11 | 2731 | 139 | 59 | 74 | 97 |
| ***NUDT17*** | nd | 26 | 58 | nd | 2202 | 76 | nd | 52 | 45 |
| ***NUDT17*** | nd | 68 | 98 | nd | 3343 | 175 | 27 | 57 | 63 |
| ***NUDT17*** | nd | 63 | 108 | nd | 3100 | 181 | nd | 48 | 62 |
| ***NUDT17*** | nd | 79 | 53 | nd | 3177 | 159 | nd | 70 | 73 |
| ***NUDT17*** | nd | 93 | 126 | nd | 4700 | 129 | nd | 99 | 116 |
| ***NUDT17*** | nd | 135 | 169 | 17 | 3975 | 220 | 193 | 101 | 73 |
| ***NUDT17*** | nd | 79 | 111 | nd | 2737 | 138 | 50 | 72 | 26 |
| ***NUDT18*** | nd | 19 | 34 | nd | 1387 | 68 | 35 | nd | 42 |
| ***NUDT18*** | nd | 102 | 141 | nd | 4796 | 129 | nd | 79 | 93 |
| ***NUDT18*** | nd | 156 | 144 | nd | 5563 | 145 | 52 | 79 | 68 |
| ***NUDT18*** | nd | 79 | 76 | 16 | 3648 | 106 | 96 | 51 | 82 |
| ***NUDT18*** | nd | 43 | 58 | 11 | 2240 | 75 | 32 | nd | nd |
| ***NUDT18*** | nd | 96 | 84 | 12 | 2702 | 113 | 90 | 133 | 91 |
| ***NUDT21*** | nd | 85 | 83 | 10 | 2639 | 170 | 38 | 61 | 63 |
| ***NUDT21*** | nd | 65 | 81 | nd | 3485 | 175 | nd | 59 | 64 |
| ***NUDT21*** | nd | 0 | 0 | nd | 790 | 56 | 53 | 35 | nd |
| ***NUDT21*** | nd | 46 | 49 | nd | 3007 | 123 | nd | 54 | 56 |
| ***NUDT21*** | nd | 39 | 45 | 11 | 1964 | 96 | 36 | 21 | nd |
| ***NUDT21*** | nd | 56 | 80 | 11 | 2610 | 177 | nd | 55 | 57 |
| ***NUDT12*** | nd | 115 | 111 | 16 | 5281 | 214 | 28 | 72 | nd |
| ***NUDT12*** | nd | 46 | 99 | nd | 2637 | 119 | nd | 81 | nd |
| ***NUDT12*** | nd | 92 | 85 | 16 | 2880 | 97 | 61 | 79 | nd |
| ***NUDT12*** | nd | 75 | 87 | nd | 2979 | 201 | 60 | 65 | 18 |
| ***NUDT12*** | nd | 85 | 61 | 13 | 2521 | 134 | 60 | 67 | nd |
| ***NUDT13*** | nd | 89 | 122 | 7 | 2004 | 69 | 62 | 69 | 20 |
| ***NUDT13*** | nd | 117 | 102 | nd | 1869 | 65 | nd | 64 | 23 |
| ***NUDT13*** | nd | 73 | 84 | 7 | 1319 | 50 | 28 | 36 | 59 |
| ***NUDT13*** | nd | 124 | 156 | 10 | 3269 | 104 | 30 | 73 | 47 |
| ***NUDT13*** | 52 | 108 | 165 | 14 | 3719 | 103 | 65 | 103 | 94 |
| ***NUDT13*** | nd | 43 | 49 | nd | 649 | nd | 65 | nd | nd |
| ***NUDT16*.1** | nd | 59 | 107 | nd | 2619 | 135 | nd | 45 | 47 |
| ***NUDT16*.1** | nd | 40 | 66 | nd | 1822 | 98 | 40 | 23 | nd |
| ***NUDT16*.1** | nd | 47 | 54 | nd | 1640 | 73 | 26 | 50 | 32 |
| ***NUDT16*.1** | nd | 69 | 76 | nd | 1980 | 88 | 42 | 54 | 76 |
| ***NUDT16*.1** | nd | 40 | 32 | 4 | 1587 | 58 | nd | 29 | nd |
| ***NUDT16*.2** | nd | 57 | 107 | 11 | 1629 | 66 | 27 | 69 | 86 |
| ***NUDT16*.2** | nd | 50 | 80 | 11 | 1661 | 76 | 58 | 45 | 47 |
| ***NUDT16*.2** | nd | 39 | 35 | nd | 860 | nd | 38 | nd | nd |
| ***NUDT16*.2** | nd | 122 | 169 | 23 | 3108 | 93 | nd | 68 | 58 |
| ***NUDT16*.2** | nd | 92 | 137 | 10 | 2254 | 82 | 72 | 40 | 37 |
| ***NUDT16*.2** | nd | 100 | 111 | 11 | 1921 | 45 | 94 | 22 | nd |
| ***NUDT16*.2** | nd | 84 | 117 | 12 | 2137 | 109 | 45 | 44 | 21 |
| ***NUDT4*** | nd | 59 | 92 | 13 | 1971 | 122 | nd | 32 | 53 |
| ***NUDT4*** | nd | 47 | 64 | 6 | 1922 | 54 | 37 | 28 | 28 |
| ***NUDT4*** | nd | 91 | 117 | nd | 2114 | 93 | 43 | 34 | 25 |
| ***NUDT4*** | nd | 35 | 51 | nd | 2331 | 70 | 33 | 37 | 38 |
| ***NUDT4*** | nd | 60 | 67 | 6 | 1874 | 35 | 61 | 29 | 26 |
| ***NUDT4*** | nd | 84 | 83 | 8 | 1618 | 76 | 45 | 36 | 24 |

Table S4: (PP-)InsP values in pmol g^-1^ FW for Arabidopsis grown on peat-based substrate.

|  | **InsP_8_** | **4/6-InsP_7_** | **5-InsP_7_** | **1/3-InsP_7_** | **InsP_6_** | **1/3OH-InsP_5_** | **InsP_5_ others** | **InsP_4-1_** | **InsP_4-2_** |
| --- | --- | --- | --- | --- | --- | --- | --- | --- | --- |
| **WT** | nd | 125 | 133 | 33 | 18998 | 111 | 137 | 256 | 117 |
| **WT** | nd | 103 | 176 | 37 | 25107 | 137 | 189 | 208 | 109 |
| **WT** | nd | 199 | 330 | 50 | 26717 | 163 | 179 | 228 | 99 |
| **WT** | nd | 188 | 275 | 42 | 28691 | 197 | 343 | 368 | 240 |
| **WT** | 5 | 19 | 20 | 6 | 2859 | 18 | 20 | 24 | 11 |
| **WT** | nd | 278 | 243 | 55 | 37325 | 239 | 267 | 279 | 134 |
| **Line 1 *nudt12/13/16*** | nd | 109 | 192 | 77 | 20186 | 111 | 187 | 162 | 65 |
| **Line 1 *nudt12/13/16*** | nd | 114 | 167 | 63 | 17402 | 117 | 106 | 138 | 94 |
| **Line 1 *nudt12/13/16*** | nd | 220 | 246 | 85 | 25156 | 66 | 185 | 255 | nd |
| **Line 1 *nudt12/13/16*** | nd | 205 | 148 | 40 | 24065 | 120 | 135 | 183 | 90 |
| **Line 1 *nudt12/13/16*** | nd | 117 | 88 | 60 | 20428 | 89 | 75 | 115 | 72 |
| **Line 1 *nudt12/13/16*** | nd | 91 | 117 | 40 | 20387 | 108 | 181 | 95 | 90 |
| **Line 2 *nudt12/13/16*** | nd | 114 | 182 | 54 | 17570 | 89 | 151 | 148 | 110 |
| **Line 2 *nudt12/13/16*** | nd | 152 | 189 | 50 | 19504 | 135 | 107 | 288 | nd |
| **Line 2 *nudt12/13/16*** | nd | 212 | 212 | 57 | 20917 | 107 | 191 | 298 | nd |
| **Line 2 *nudt12/13/16*** | nd | 158 | 157 | 62 | 23175 | 101 | 159 | 314 | nd |
| **Line 2 *nudt12/13/16*** | nd | 145 | 199 | 44 | 26691 | 140 | nd | 198 | nd |

Table S5: List of differentially expressed genes (DEGs) and no-DEGs related to P. Upregulated and downregulated genes have a |log_2_FC| > 1 and Q-values < 0.05. Non-significant genes have Q-values > 0.05.

|  | **Gene name** | **Gene ID** | **Log_2_FC** |
| --- | --- | --- | --- |
| **Upregulated** | *SDI1* | AT4G14070 | 3.07 |
|  | *AAE15* | AT3G47340 | 2.80 |
|  | *ASN1* | AT2G18700 | 2.17 |
|  | *TPS9* | AT5G48850 | 1.67 |
|  | *TPS11* | AT1G23870 | 1.64 |
|  | *SDI2* | AT1G04770 | 1.11 |
|  |  |  |  |
| **Downregulated** | *DIC2* | AT5G04340 | -3.30 |
|  | *ZAT6* | AT4G24570 | -3.06 |
|  | *GLTP* | AT4G39670 | -2.98 |
|  | *TIR-NBS9* | AT1G72920 | -2.37 |
|  |  | AT4G36010 | -2.03 |
|  | *ACS6* | AT4G11280 | -1.99 |
|  | *CaLB1* | AT4G34150 | -1.85 |
|  | *DIC1* | AT2G22500 | -1.84 |
|  | PR5 | AT1G75040 | -1.68 |
|  | *MYB62* | AT1G68320 | -1.62 |
|  | *PAP1* | AT2G01180 | -1.48 |
|  | *ATL80* | AT1G20823 | -1.4 |
|  | *CaLB domain* | AT3G16510 | -1.38 |
|  | *SOT17* | AT1G18590 | -1.28 |
|  | *GPAT6* | AT2G38110 | -1.21 |
|  |  |  |  |
| **Non-significant** | *SPX1* | AT5G20150 |  |
|  | *BAH1* | AT1G02860 |  |
|  | *WRKY6* | AT1G62300 |  |
|  | *VPT3* | AT4G22990 |  |
|  | *PHB* | AT2G34710 |  |
|  | *PHT4;5* | AT5G20380 |  |
|  | *LPR1* | AT1G23010 |  |
|  | *BAK1* | AT4G33430 |  |
|  | *PHR2* | AT2G47590 |  |
|  | *PAP10* | AT2G16430 |  |
|  | *SPX3* | AT2G45130 |  |
|  | *STOP1* | AT1G34370 |  |
|  | *SIZ1* | AT5G60410 |  |
|  | *PHL4* | AT2G20400 |  |
|  | *SPX2* | AT2G26660 |  |
|  | *PHR1* | AT4G28610 |  |
|  | *PEPR2* | AT1G17750 |  |
|  | *PBL12* | AT2G26290 |  |
|  | *PHO2* | AT2G33770 |  |
|  | *VPT1* | AT1G63010 |  |
|  | *MYB62* | AT1G68320 |  |
|  | *CERK1* | AT3G21630 |  |
|  | *PHT1;4* | AT2G38940 |  |
|  | *GLP1* | AT1G72610 |  |
|  | *bHLH050* | AT1G73830 |  |
|  | *VIH2* | AT3G01310 |  |
|  | *EAL1* | AT4G37650 |  |
|  | *PHT3;1* | AT5G14040 |  |
|  | *ALS3* | AT2G37330 |  |
|  | *MRP5* | AT1G04120 |  |
|  | *ALMT1* | AT1G08430 |  |
|  | *CLV2* | AT1G65380 |  |
|  | *LPR2* | AT1G71040 |  |
|  | *PAP12* | AT2G27190 |  |
|  | *PHT5* | AT2G32830 |  |
|  | *MOR1* | AT2G35630 |  |
|  | *SPDT* | AT3G15990 |  |
|  | *RALF23* | AT3G16570 |  |
|  | *PHO1* | AT3G23430 |  |
|  | *PHL2* | AT3G24120 |  |
|  | *PHT2;1* | AT3G26570 |  |
|  | *PHL3* | AT4G13640 |  |
|  | *ITPK2* | AT4G33770 |  |
|  | *IPS2* | AT5G03545 |  |
|  | *WRKY75* | AT5G13080 |  |
|  | *VIH1* | AT5G15070 |  |
|  | *SPX4* | AT5G15330 |  |
|  | *ITPK1* | AT5G16760 |  |
|  | *SEC12* | AT2G01470 |  |
|  | *PDR2* | AT5G23630 |  |

Table S6: Most highly downregulated and upregulated genes, defined as |log_2_FC| > 3.

|  | **Gene name** | **Gene ID** | **Log_2_FC** |
| --- | --- | --- | --- |
| **Downregulated** | *PAO2* | AT2G43020 | -9,57 |
|  | U-box E3 ubiquitin | AT3G02840 | -4,09 |
|  | *CMF4* | AT1G63820 | -3,88 |
|  | *ATS40-2* | AT5G45630 | -3,87 |
|  | *RAS1* | AT1G09950 | -3,83 |
|  | *WRKY40* | AT1G80840 | -3,64 |
|  | *DVL10* | AT4G13395 | -3,64 |
|  | *ERF022* | AT1G33760 | -3,63 |
|  | *ERF11* | AT1G28370 | -3,53 |
|  |  | AT4G29780 | -3,52 |
|  | *IDL7* | AT3G10930 | -3,49 |
|  | *CCR4* | AT5G47850 | -3,44 |
|  | *DIC2* | AT4G24570 | -3,29 |
|  | *DHYPRP1* | AT4G22470 | -3,27 |
|  | *DTX50* | AT5G52050 | -3,10 |
|  |  | AT5G16200 | -3,07 |
|  | *ZAT6* | AT5G04340 | -3,06 |
|  | *ZAT10* | AT1G27730 | -3,01 |
|  | *ERF6* | AT4G17490 | -3,01 |
|  |  |  |  |
| **Upregulated** | *SDI1* | AT5G48850 | 3,06 |
|  | *IRONMAN 3* | AT2G30766 | 3,71 |
|  |  | AT2G05540 | 3,78 |
|  | *LTP3* | AT5G59320 | 4,45 |
|  | *IRP6* | AT5G05250 | 4,46 |
|  |  | AT5G35935 | 5,64 |

Table S7: List of differentially expressed genes (DEGs) and no-DEGs related to Fe. Upregulated and downregulated genes have a |log_2_FC| > 1 and Q-values < 0.05. Non-significant genes have Q-values > 0.05.

|  | **Gene name** | **Gene ID** | **Log_2_FC** |
| --- | --- | --- | --- |
| **Upregulated** | *IRP6* | AT5G05250 | 4.46 |
|  | *IRONMAN 3* | AT2G30766 | 3.72 |
|  |  |  |  |
| **Downregulated** | *ZAT12* | AT5G35735 | -2.66 |
|  | *FER1* | AT5G01600 | -2.02 |
|  | *HYP1* | AT5G59820 | -1.30 |
|  |  |  |  |
| **Non- significant** | *NRAMP2* | AT1G47240 |  |
|  | *NAS1* | AT5G04950 |  |
|  | *NRAMP1* | AT1G80830 |  |
|  | *DEG18* | AT4G12980 |  |
|  | *CRR* | AT3G25290 |  |
|  | *YSL6* | AT3G27020 |  |
|  | *BHLH115* | AT1G51070 |  |
|  | *AHA2* | AT4G30190 |  |
|  | *BTS* | AT3G18290 |  |
|  | *AHA8* | AT3G42640 |  |
|  | *BHLH38* | AT3G56970 |  |
|  | *YSL2* | AT5G24380 |  |
|  | *AHA10* | AT1G17260 |  |
|  | *LPR1* | AT1G23010 |  |
|  | *IRT3* | AT1G60960 |  |
|  | *AIR12* | AT3G07390 |  |
|  | *COSY* | AT1G28680 |  |
|  | *VTL1* | AT1G21140 |  |
|  | *MYB10* | AT3G12820 |  |
|  | *BHLH34* | AT3G23210 |  |
|  | *CIPK11* | AT2G30360 |  |
|  | *AHA1* | AT2G18960 |  |
|  | *VTL2* | AT1G76800 |  |
|  | *YSL5* | AT3G17650 |  |
|  | CYBDOMs | AT5G54830 |  |
|  | *YSL3* | AT5G53550 |  |
|  | *NAS4* | AT1G56430 |  |
|  | *AHA3* | AT5G57350 |  |
|  | *IRONMAN 2* | AT1G47395 |  |
|  | *BHLH121* | AT3G19860 |  |
|  | *BHLH29* | AT2G28160 |  |
|  | *BHLH105* | AT5G54680 |  |
|  | *PYE* | AT3G47640 |  |
|  | *AHA4* | AT3G47950 |  |
|  | *BHLH100* | AT2G41240 |  |
|  | CYBDOMs | AT5G47530 |  |
|  | *YSL4* | AT5G41000 |  |
|  | *YSL1* | AT4G24120 |  |
|  | *AHA5* | AT2G24520 |  |
|  | *AHA9* | AT1G80660 |  |
|  | *AHA11* | AT5G62670 |  |
|  | *YSL8* | AT1G48370 |  |
|  | *YSL7* | AT1G65730 |  |
|  | *IRONMAN 1* | AT1G47400 |  |
|  | *NRAMP6* | AT1G15960 |  |
|  | *BHLH39* | AT3G56980 |  |
|  | *NAS3* | AT1G09240 |  |
|  | *BTSL2* | AT1G18910 |  |
|  | *LPR2* | AT1G71040 |  |
|  | *BTSL1* | AT1G74770 |  |
|  | *NRAMP3* | AT2G23150 |  |
|  | CYBDOMs | AT3G07570 |  |
|  | *FRD3* | AT3G08040 |  |
|  | *F6'H1* | AT3G13610 |  |
|  | *VTL5* | AT3G25190 |  |
|  | *PDR9* | AT3G53480 |  |
|  | *MTP8* | AT3G58060 |  |
|  | *CYBDOMs* | AT3G59070 |  |
|  | *AHA7* | AT3G60330 |  |
|  | *BHLH104* | AT4G14410 |  |
|  | CYBDOMs | AT4G17280 |  |
|  | *NRAMP5* | AT4G18790 |  |
|  | *IRT1* | AT4G19690 |  |
|  | *BHLH101* | AT5G04150 |  |
|  | *ZIF1* | AT5G13740 |  |
|  | *NRAMP4* | AT5G67330 |  |

Table S8: List of primers used in this study.

| **Cloning primer** |  |  |
| --- | --- | --- |
| **Target gene** | **Objective** | **Primer sequence 5’-3’** |
| *NUDT4* | attb1 cDNA | AAAAAGCAGGCTTC ATGACAGGGTTCTCTGTGTC |
|  | attb1 promoter | AAAAAGCAGGCTTC GTCCGACTTTAAAGAGAAATTGAGG |
|  | attb2 stop | AGAAAGCTGGGTC TCAGTTCCCACTTTCATCATCG |
|  | attb2 no stop | AGAAAGCTGGGTC GTTCCCACTTTCATCATCGTC |
|  | attb2 no stop V5 | ACCTCCTCCAGATCCGTTCCCACTTTCATCATCGTC |
|  | genotyping | F:AAAAAGCAGGCTTC GTCCGACTTTAAAGAGAAATTGAGG  R: AGAAAGCTGGGTC GTTCCCACTTTCATCATCGTC  Seq:ACTCTTTCTCTCTTGTTCTG |
| *NUDT17* | attb1 cDNA | AAAAAGCAGGCTTC ATGGGTGTTGAGAAAATGGTG |
|  | attb1 promoter | AAAAAGCAGGCTTC GTCGTATTGTATGTTCCATGC |
|  | attb2 stop | AGAAAGCTGGGTC TCAACACATTGTTTCAATAGAGATTG |
|  | attb2 no stop | AGAAAGCTGGGTC ACACATTGTTTCAATAGAGATTGAC |
|  | attb2 no stop V5 | ACCTCCTCCAGATCCACACATTGTTTCAATAGAGATTGAC |
|  | genotyping | F: AAAAAGCAGGCTTC GTCGTATTGTATGTTCCATGC  R: AGAAAGCTGGGTC TCAACACATTGTTTCAATAGAGATTG  Seq:CATCAGACCCTTCTCTTCTC |
| *NUDT18* | attb1 cDNA | AAAAAGCAGGCTTC ATGGTGTGTTTGGTCTCCC |
|  | attb1 promoter | AAAAAGCAGGCTTC GAGCTTCTTCTAGATCGAGCTG |
|  | attb2 stop | AGAAAGCTGGGTC TCAGTAGATAGAGATCAGTGGAAG |
|  | attb2 no stop | AGAAAGCTGGGTC GTAGATAGAGATCAGTGGAAGGTTC |
|  | attb2 no stop V5 | ACCTCCTCCAGATCCGTAGATAGAGATCAGTGGAAGGTTC |
|  | genotyping | F: GGATAAGCCGCAAGGAGG  R: TTTCCAAGTTGTCTCTGCACC  Seq:CTCACCCCATCATAACTTC |
| *NUDT21* | attb1 cDNA | AAAAAGCAGGCTTCATGATTTCTCTATTCATCTCAAACTTTTC |
|  | attb1 promoter | AAAAAGCAGGCTTC CTCCGTAAATACCGTGTTGG |
|  | attb2 stop | AGAAAGCTGGGTC TTATTGGGTCTGGCATTTCC |
|  | attb2 no stop | AGAAAGCTGGGTC TTGGGTCTGGCATTTCC |
|  | attb2 no stop V5 | ACCTCCTCCAGATCCTTGGGTCTGGCATTTCC |
|  | genotyping | F: AAAAAGCAGGCTTC CTCCGTAAATACCGTGTTGG  R: AGAAAGCTGGGTC TTATTGGGTCTGGCATTTCC  Seq:ATAAAGACGCTCGCAAAC |
| *NUDT12* | attb1 cDNA | AAAAAGCAGGCTTC ATGTCGGTTCTTTCTTCTCG |
|  | attb1 promoter | AAAAAGCAGGCTTC CCCTTCTGTTTCGTACATGC |
|  | attb2 stop | AGAAAGCTGGGTC CTAGTTAACTACAAAACAGTACCAAGG |
|  | attb2 no stop | AGAAAGCTGGGTCGTTAACTACAAAACAGTACCAAGG |
|  | attb2 no stop V5 | ACCTCCTCCAGATCCGTTAACTACAAAACAGTACCAAGG |
|  | qPCR | F: AACTCGAGGATTGGCCAGAGCGA  R: CCGACAAAGCTCCAACGCTTCT |
|  | genotyping | F: CCCTTCTGTTTCGTACATGC  R: CTAGTTAACTACAAAACAGTACCAAGG  Seq: CCCTGACTGTCTCATCATTC |
| *NUDT13* | attb1 cDNA | AAAAAGCAGGCTTC ATGTCGAATCTTTCTGCAAG |
|  | attb1 promoter | AAAAAGCAGGCTTC GGGGACATTTGTTCTACACAG |
|  | attb2 stop | AGAAAGCTGGGTC TTAGACTACAAAGCAGTAGCG |
|  | attb2 no stop | AGAAAGCTGGGTCGACTACAAAGCAGTAGCGAG |
|  | attb2 no stop V5 | ACCTCCTCCAGATCCGACTACAAAGCAGTAGCGAG |
|  | qPCR | F: AGGCTGGTGAAAGATGAAGAAGA  R: TCCCATCCTCCCTTTGGGAA |
|  | genotyping | F: ATGTCGAATCTTTCTGCAAG  R: TTAGACTACAAAGCAGTAGCG  Seq: TTAGACTACAAAGCAGTAGCG |
| *NUDT16 both* | attb1 promoter | AAAAAGCAGGCTTC CAATTACCTGCGATCTCTCTCTG |
|  | attb2 stop | AGAAAGCTGGGTC TCAATGTTCACCAGTTATCTCCTCTCC |
|  | attb2 NS | AGAAAGCTGGGTC ATGTTCACCAGTTATCTCCTCTCC |
|  | attb2 NS V5 | ACCTCCTCCAGATCCATGTTCACCAGTTATCTCCTCTCC |
|  | qPCR | F: CGCCGGGTGTATTCCGTTTA  R: AGGTCCACTAGACGAGCTGA |
|  | genotyping | F: CAATTACCTGCGATCTCTCTCTG  R: ATGTTCACCAGTTATCTCCTCTCC  Seq: AGCTATAGACAGACACACCC |
| *NUDT16.1* | attb1 cDNA | AAAAAGCAGGCTTCATGTGTGATTTGGTCGCGCG |
|  | qPCR | F: TGTCTCCAAGCTGGGTGTTT  R: CGCGACCAAATCACACATGA |
| *NUDT16.2* | attb1 cDNA | AAAAAGCAGGCTTCATGGTAGAGCAGCGGTACGAG |
| *MoNUDT^E79Q^* | mutagenesis forward | CTGGGAAACCTGTGTGCGCCGTCAAGCGCGAGAAGAAGGCGGATTTACTTTG |
| *MoNUDT^E79Q^* | mutagenesis reverse | CAAAGTAAATCCGCCTTCTTCTCGCGCTTGACGGCGCACACAGGTTTCCCAG |
|  |  |  |
| General | attb1 adapter | GGGGACAAGTTTGTACAAAAAAGCAGGCTTC |
|  | attb1 adapter | GGGGACCACTTTGTACAAGAAAGCTGGGTC |
|  | attb2 V5 adapter | GGGGACCACTTTGTACAAGAAAGCTGGGTCTTAC  GTAGAATCGAGACCGAGGAGAGGGTTAGGGATA  GGCTTACCTCCTCCAGATCC |
|  |  |  |
| **Line/Plasmid** | **Primer Sequence** | |
| SALK_102051.27.90.x | F: CAACAACGTTTGGGCTTCTAG  R: ATTGTGAATTTGAACAACGGC | |
| SAIL_1211_A06 | F: TTGGTGGTTTACGAGTTGACC  R: TCAACACGTTATTCCAAATTGC | |
| SAIL_500_D10 | F: GAAAAATTGCTCCACACTTGC  R: TGAAGAAGAACGTACCCAACG | |
| SALKseq_117563.1 | F: AAACCAAACCGAAATCCAAAC  R: AGATCCTCCTTCCCTTTAGCC | |
| SALK_031788.56.00.x | F: TCACCCACTTGCTTGCTACTC  R: TGCTTTTGTATTGCCATTTCC | |
| SALKseq_132932.101 | F: AAATACCGTGTTGGCATGAAC  R: TGCTTTTGTATTGCCATTTCC | |
| oRU906 | GAGTCTATGATCAAGTAATTATGC | |
| oRU908 | GCTTGCATGCCTGCAGGTCGACTCT | |
| oRU385 | CAACGCGTTGGGAGCTCTCCCATATG | |
| LBa1 SALK | TGGTTCACGTAGTGGGCC | |
| LB2 SAIL | GCTTCCTATTATATCTTCCCAAATTACCAATACA | |
|  |  | |
| **qPCR** | **Primer Sequence 5’-3’** | |
| *ACTIN2* | F: GACCAGCTCTTCCATCGAGAA  R: CAAACGAGGGCTGGAACAAG | |
| *UBQ2* | F: AGACGAACGCAAAGATGCAG  R: CCGGCGAAGATCAACCTCTG | |

Table S9: List of primers used to generate sgRNAs for the *Nudix-type* (*NUDT)* hydrolase knockout mutants. Overhangs for oligo annealing are highlighted.

| **Target gene** | **gRNA** | **Forward primer** | **Reverse primer** |
| --- | --- | --- | --- |
| *NUDT17* | sgRNA1 | **ATTG** CAGTTTCAGAGATACAACAA | **AAAC** TTGTTGTATCTCTGAAACTG |
| *NUDT17* | sgRNA2 | **GTCA** CCACGCCTTAATGTTCCCCA | **AAAC** TGGGGAACATTAAGGCGTGG |
| *NUDT18* | sgRNA1 | **ATTG** CAATCCCAAAGATACAACAA | **AAAC** TTGTTGTATCTTTGGGATTG |
| *NUDT18* | sgRNA2 | **GTCA** TCACGCTCTGATGTTCCCAA | **AAAC** TTGGGAACATCAGAGCGTGA |
| *NUDT21* | sgRNA1 | **ATTG** CCCATCGAAGAAGATCGTAC | **AAAC** GTACGATCTTCTTCGATGGG |
| *NUDT21* | sgRNA2 | **GTCA** TATAGATACAAGAAACACGG | **AAAC** CCGTGTTTCTTGTATCTATA |
| *NUDT4* | sgRNA1 | **ATTG** GAAGATGGGCGACAAATACG | **AAAC** CGTATTTGTCGCCCATCTTC |
| *NUDT4* | sgRNA2 | **GTCA** GGAGACGGATGAATCAATGG | **AAAC** CCATTGATTCATCCGTCTCC |
| *NUDT12* | sgRNA1 | **ATTG** TTGGCAGATTCATCAAATGT | **AAAC** ACATTTGATGAATCTGCCAA |
| *NUDT12* | sgRNA2 | **GTCA** ATATTATTATCAGGGAGGAT | **AAAC** ATCCTCCCTGATAATAATAT |
| *NUDT13* | sgRNA1 | **ATTG** ATGACAGATGCATTCCGTAT | **AAAC** ATACGGAATGCATCTGTCAT |
| *NUDT13* | sgRNA2 | **GTCA** CGTGAAGCTATGGAAGAAGC | **AAAC** GCTTCTTCCATAGCTTCACG |
| *NUDT16* | sgRNA1 | **ATTG** TCGTCTCCAGCAGCGGTACG | **AAAC** CGTACCGCTGCTGGAGACGA |
| *NUDT16* | sgRNA2 | **GTCA** GAATGATGAGACAGTCAGGG | **AAAC** CCCTGACTGTCTCATCATTC |
